# A quantum-inspired cross-attention fusion network for multi-class eye disease classification from retinal fundus images

**DOI:** 10.3389/fopht.2026.1975827

**Published:** 2026-10-16

**Authors:** Abinash P, Krishnamoorthy N

**Affiliations:** School of Computer Science Engineering and Information Systems (SCORE), Vellore Institute of Technology, Vellore, India

**Keywords:** cross-attention fusion, diabetic retinopathy, EfficientNet, explainable artificial intelligence, external validation, fundus image classification, glaucoma, Grad-CAM++

## Abstract

Automated screening of blinding eye diseases from colour fundus photographs is an active area of medical image analysis, yet many reported systems are validated on a single dataset and offer limited insight into their internal decision process. This study introduces QICAF-Net, a dual-branch deep learning framework that couples a Swin Transformer global-context branch with an EfficientNet local-lesion branch and fuses their representations through a novel Quantum-Inspired Cross-Attention Fusion (QICAF) module. The QICAF module reformulates cross-attention using the mathematical formalism of quantum states: token features are amplitude-encoded as complex unit vectors, transformed by a learnable norm-preserving unitary gate, compared through a fidelity-inspired similarity, normalised by a Born-rule measurement rule, and combined through an entanglement-inspired bilinear interaction. All operations are executed as classical linear algebra on conventional graphics hardware; no physical quantum computer and no quantum speed-up are claimed. The model was trained and evaluated on a balanced four-class retinal dataset of 4,217 fundus images spanning cataract, diabetic retinopathy (DR), glaucoma and normal categories. Under stratified five-fold cross-validation with six-view test-time augmentation (TTA), QICAF-Net attained a macro-F1 of 0.9358 ± 0.0113, an accuracy of 0.9367 ± 0.0112 and a macro one-versus-rest area under the receiver operating characteristic curve (AUC) of 0.9914 ± 0.0023; a three-seed evaluation of the full model gave a macro-F1 of 0.9420 ± 0.0071. An 18-run multi-seed component ablation (six fusion configurations × three seeds) confirmed that the quantum-inspired components achieve accuracy statistically indistinguishable from strong classical fusion baselines, and a paired three-seed significance test between QICAF-Net and a matched dual-branch concatenation model returned no statistically significant accuracy difference (paired t-test p = 0.887; Wilcoxon p = 1.000). Against five single-backbone baselines under an identical preprocessing and TTA protocol, QICAF-Net was competitive but did not exceed the strongest backbones (Swin-Tiny 0.9583, ViT-Small 0.9487). The principal contribution of the module is therefore a novel and interpretable formulation rather than a raw accuracy gain. External validation on a heterogeneous independent cohort (N = 3,907) showed heterogeneous cross-dataset transfer — better preserved for cataract and normal and substantially degraded for glaucoma; under an observed external-domain shift the diabetic-retinopathy sensitivity fell to 39.61%, and under colour-constancy correction with six-view TTA the external accuracy was 0.8741 (95% CI 0.8633–0.8841) and the macro-F1 0.7188 (95% CI 0.6969–0.7398). Two complementary explainability methods together with visualisation of the QICAF attention indicated that the model attends to clinically plausible regions, most strongly the optic nerve head for glaucoma. Calibration, quantitative explanation-faithfulness (deletion/insertion) and profiler-based computational analyses were additionally performed. QICAF-Net is thus presented as an interpretable and transparently evaluated framework whose accuracy is on par with established fusion strategies while contributing a new attention formulation to the retinal imaging literature.

## 1 Introduction

Vision-threatening eye disease is one of the largest global health burdens, and a substantial proportion of the associated blindness is avoidable when the underlying condition is detected early. Diabetic retinopathy (DR), glaucoma and cataract together account for a major share of irreversible and reversible visual impairment worldwide, and all three can be assessed from colour fundus photography, a low-cost and widely deployed imaging modality (1). The clinical challenge is that early disease is subtle: microaneurysms and small haemorrhages in diabetic retinopathy, mild cupping of the optic nerve head in glaucoma and faint lens opacity in cataract are easily missed, particularly where trained graders are scarce (9). Automated decision support that is both accurate and interpretable therefore has clear value for large-scale screening.

Deep learning has transformed retinal image analysis over the past decade. Convolutional neural networks first demonstrated ophthalmologist-level performance for diabetic retinopathy detection (6), and subsequent work extended the paradigm to multi-disease classification, lesion localisation and severity grading (4). More recently, vision transformers and hybrid convolutional-transformer designs have been adopted because they capture long-range spatial dependencies that pure convolutional models struggle to represent, which is relevant for structures such as the optic disc and the global vascular pattern (16). Attention mechanisms are now a standard component of high-performing retinal models, and cross-attention in particular has been used to let features from different sources or scales inform one another (2).

Two limitations recur across the literature and motivate the present work. First, most reported systems are trained and tested on a single dataset, and the small number of studies that perform external validation frequently report performance that equals or even exceeds internal performance, a pattern that is difficult to reconcile with genuine distribution shift and that raises questions about the difficulty of the external test set. Rigorous, transparent cross-dataset evaluation remains uncommon (10). Second, the fusion of complementary representations is often performed by simple concatenation or summation, and the added value of more elaborate fusion is rarely isolated through controlled ablation and statistical testing. Where novel modules are proposed, the evidence that they outperform trivial baselines is frequently limited to a single split.

This paper addresses both limitations. The methodological contribution is a Quantum-Inspired Cross-Attention Fusion (QICAF) module that reformulates the cross-attention between a global transformer branch and a local convolutional branch using the mathematical language of quantum states. Feature vectors are amplitude-encoded as complex unit vectors, evolved by a learnable norm-preserving unitary gate, compared through a fidelity-inspired similarity, converted to attention weights by a Born-rule measurement rule, and finally combined through an entanglement-inspired bilinear interaction. It is important to state clearly at the outset that these operations are realised entirely as classical linear algebra on conventional hardware. The module borrows the formalism of quantum mechanics as an inductive bias for designing attention; it does not run on a quantum computer, and no quantum computational advantage or speed-up is claimed (24). This framing is deliberate and is maintained throughout the paper.

The evaluation contribution is a deliberately conservative and complete experimental protocol. The proposed model is assessed with stratified five-fold cross-validation, compared against five standard single-backbone baselines, subjected to a cumulative component ablation, and, crucially, compared against a matched dual-branch concatenation model using a paired three-seed significance test. The framework is then externally validated on an independent cohort, and interpreted with two complementary explainability methods together with a direct visualisation of the learned QICAF attention. Where an experimental outcome is nuanced, for example the observation that the quantum-inspired formulation matches rather than exceeds classical fusion in accuracy, the finding is reported openly and its scientific explanation is given, rather than being obscured.

The main contributions of this study are summarised as follows:

1. A novel quantum-inspired cross-attention fusion module is proposed, in which amplitude encoding, a unitary phase gate, fidelity-inspired similarity, Born-rule normalisation and entanglement-inspired fusion are combined to form an interpretable cross-attention mechanism between a global transformer branch and a local convolutional branch.
2. A dual-branch architecture, QICAF-Net, is constructed around this module using a Swin Transformer global branch and an EfficientNet local branch, and is trained with a numerically stable class-weighted focal objective, exponential moving averaging and mixed-sample augmentation.
3. A rigorous evaluation is performed comprising five-fold cross-validation, five single-backbone baselines, a six-configuration cumulative ablation, and a paired three-seed significance test that transparently establishes the accuracy relationship between the quantum-inspired module and matched classical fusion.
4. An independent external validation is conducted, including characterization of diabetic-retinopathy performance degradation under external-domain shift and an exploratory colour-constancy adaptation analysis, together with two explainability methods and a direct visualisation of the QICAF attention to support clinical interpretability.

The remainder of the paper is organised as follows. Section 2 reviews related work on deep learning for retinal disease classification, attention and fusion mechanisms, quantum-inspired learning and explainability. Section 3 describes the dataset, model configuration, complexity, implementation and evaluation metrics. Section 4 presents the proposed methodology in detail, including the mathematical formulation of the QICAF module and the overall algorithm. Section 5 reports and discusses the experimental results. Sections 6 to 9 address clinical applicability, comparative analysis, limitations and future work, and the conclusion.

## 2 Literature survey

### 2.1 Deep learning for retinal disease classification

The application of deep learning to fundus image analysis began with binary and severity-graded diabetic retinopathy detection. Large-scale studies established that convolutional networks could match specialist graders for referable diabetic retinopathy (38), and image-based deep learning was subsequently shown to generalise across several treatable conditions (7). These results catalysed a broad research programme on multi-disease retinal classification. A framework for the early detection of multiple retinal diseases and emphasised the importance of balanced multi-class evaluation (1), while ensemble and correlation-based designs were introduced to exploit the statistical dependencies between co-occurring conditions (4). Computer-aided multi-label pipelines with graph-based inter-disease regularisation (3) and information-fusion systems that combine multiple fundus representations (5) have further advanced multi-condition detection, and artificial-intelligence screening across multiple retinal and optic-nerve diseases has been demonstrated at population scale (8). Attention-guided segmentation of the optic disc and cup has likewise improved the structural analysis on which several of these systems depend (15). Reviews of the field document a rapid migration from single-disease pipelines toward unified multi-class systems, and consistently identify the scarcity of standardised, externally validated benchmarks as a barrier to clinical translation (10).

Disease-specific studies have refined the representations most useful for each condition. For diabetic retinopathy, lesion-aware and segmentation-guided architectures improve sensitivity to microaneurysms and haemorrhages (11), dedicated microaneurysm-detection pipelines target the earliest visible lesions (12), and attention-augmented convolutional models such as convolutional block attention designs have improved severity grading (17). Related approaches based on synaptic-metaplasticity learning (18) and automated haemorrhage detection (21) further strengthen retinopathy screening, and large-scale clinical validations report automated detection of diabetic retinopathy and macular oedema at scale (13). For glaucoma, attention-based detection and joint optic disc and cup segmentation have proven effective because the discriminative signal is concentrated at the optic nerve head (14), and dedicated reviews document the maturation of machine-learning methods for this task (22). Transfer-learning and fine-tuning strategies remain competitive across all three diseases and are frequently used as strong baselines (19), including self-attention transfer designs (20) and supervised fine-tuning schemes (23). Ultra-widefield and multi-modal extensions further broaden the range of detectable conditions (28).

### 2.2 Attention and feature-fusion mechanisms

Attention mechanisms allow a network to weight the most informative parts of an input and have become integral to medical image analysis (27). In retinal imaging, cross-disease attention networks share information between related grading tasks (2), and dense-correlation networks together with transformer-based classifiers exploit inter-label structure for multi-label prediction (16). The combination of a convolutional branch, which captures fine local texture, with a transformer branch, which captures global structure, is a recurring and effective design pattern, and the manner in which the two branches are fused materially affects performance. Concatenation, summation, weighted combination and attention-based fusion have all been studied, and controlled comparisons indicate that no single fusion rule dominates across datasets. The present work contributes a new fusion rule grounded in quantum formalism and, importantly, subjects it to the paired statistical comparison against concatenation that such claims require.

### 2.3 Quantum-inspired learning

Quantum-inspired machine learning imports the mathematical structure of quantum mechanics, complex-valued state vectors, unitary evolution, measurement probabilities and entanglement, into classical models without requiring quantum hardware. Quantum-probability-inspired neural networks have been applied to document representation and classification (26), conversational emotion recognition (25) and natural language processing (24), where the density-matrix and superposition formalisms provide a principled way to represent higher-order dependencies and uncertainty. These studies motivate the hypothesis that a quantum-inspired attention formulation may offer a distinctive and interpretable inductive bias. The contribution of the present work is to translate this formalism into a cross-attention module for medical image fusion and to evaluate honestly whether it confers an accuracy benefit over classical attention, a question that prior quantum-inspired studies rarely settle with paired significance testing.

### 2.4 Explainability in medical imaging

Clinical adoption of automated systems depends on interpretability. Gradient-weighted class activation mapping and its refinement Grad-CAM++ are the most widely used visual explanation methods, and comparative studies show that they highlight clinically relevant regions across a range of modalities, while also noting that gradient-based maps can struggle to localise very small lesions such as those in early diabetic retinopathy (29). Vision-transformer explainability introduces additional considerations because attention is distributed across tokens rather than convolutional feature maps. To provide robust interpretation, the present study combines Grad-CAM++ with a gradient-free occlusion-sensitivity analysis and a direct visualisation of the QICAF attention learned by the module, so that the evidence for clinical plausibility does not rest on a single method.

### 2.5 Summary of related work

**Table 1** Summarizes representative studies across the themes above, indicating the year, the methodological approach, the task focus and the principal limitation that the present work seeks to address. The recurring gaps are the reliance on single-dataset validation, the limited isolation of the fusion contribution and the uneven treatment of interpretability.

**Table 1:** Summary of representative related work in deep learning for retinal disease classification, attention and fusion, and quantum-inspired learning.

Study	Year	Method	Focus	Key limitation
(6)	2016	Deep CNN screening	DR detection	Single disease
(2)	2020	Cross-disease attention network	DR and DME grading	Two-task only
(14)	2020	Attention-based CNN	Glaucoma detection	Single disease
(4)	2021	Dense-correlation network	Multi-label ocular	Limited interpretability
(26)	2021	Quantum-probability GNN	Document classification	Non-imaging domain
(16)	2023	Vision transformer	Multi-label retinal	Single dataset
(24)	2024	Quantum-inspired DNN	Natural language	No paired testing
(30)	2023	Ensemble deep model	Four-class eye disease	Fusion not isolated
**Proposed work**	**2026**	**Quantum-inspired dual-branch fusion**	**Four-class eye disease**	**Addressed via paired test and external validation**

Bold indicates the proposed work.

## 3 Materials and model configuration

### 3.1 Dataset description

All internal experiments used a publicly available balanced retinal fundus dataset comprising 4,217 colour images distributed across four diagnostic categories: cataract, diabetic retinopathy, glaucoma and normal (31). The images were aggregated from several established sources of high-resolution fundus photography, including the Indian Diabetic Retinopathy Image Dataset, the Ocular Recognition collection and the High-Resolution Fundus database, which contributes to intra-class diversity in camera characteristics and acquisition conditions. The class distribution is approximately balanced, containing 1,098 diabetic retinopathy, 1,074 normal, 1,038 cataract and 1,007 glaucoma images, as shown in **Figure 1**. The near-uniform distribution reduces the risk of majority-class bias, although a residual imbalance remains and is handled explicitly by the class-weighted objective described in Section 4. For external validation, two independent public datasets were used: the Ocular Disease Recognition dataset (ODIR-5K) for the cataract, glaucoma and normal classes (32), and the Messidor-2 diabetic retinopathy database for the diabetic retinopathy class (33).

**Figure 1:**
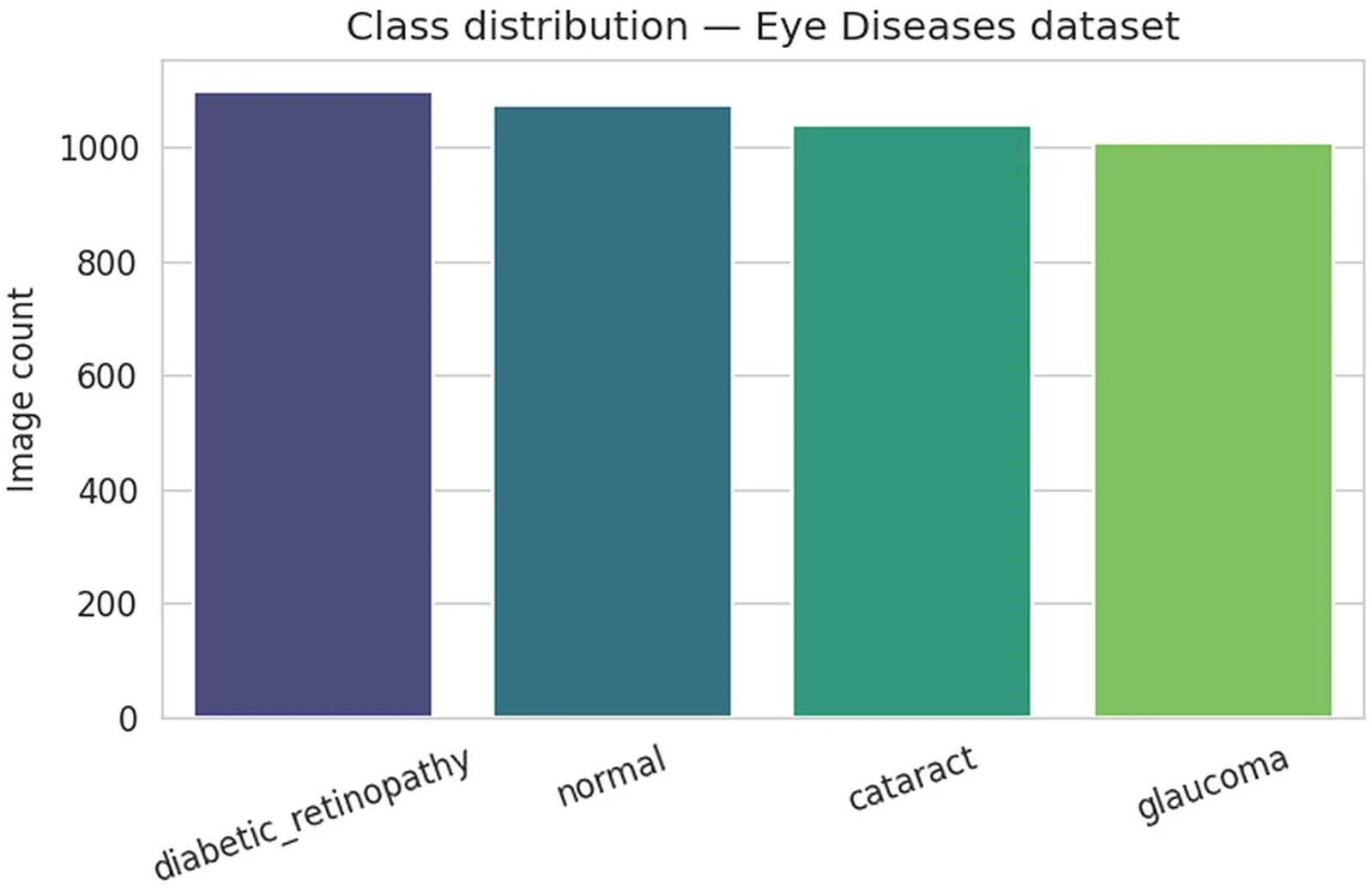
Class distribution of the four-category fundus dataset, showing an approximately balanced allocation across diabetic retinopathy, normal, cataract and glaucoma.

Each category exhibits characteristic morphology. Cataract images show diffuse lens opacity that lowers overall contrast and sharpness; diabetic retinopathy images contain microaneurysms, haemorrhages and exudates distributed across the posterior pole; glaucoma images present an enlarged cup-to-disc ratio and neuro-retinal rim thinning localised to the optic nerve head; and normal images display intact retinal structure without pathological markers. These differences motivate a dual-branch design in which a local branch captures focal lesions while a global branch captures optic-disc geometry and vascular structure.

### 3.2 Preprocessing pipeline

A four-stage preprocessing pipeline standardises the images before training, illustrated for one representative image per class in **Figure 2**. First, a circular region-of-interest crop removes the uninformative black border surrounding the fundus. Second, the image is resized to a fixed spatial resolution. Third, contrast-limited adaptive histogram equalisation is applied to the green channel, which carries the strongest vessel and lesion contrast in fundus photography. Fourth, a Gaussian-based background-subtraction normalisation (the Ben-Graham method) suppresses inter-image illumination variation. The model operates on the fully preprocessed representation, while visual explanations are overlaid on the natural-colour image for clinical readability.

**Figure 2:**
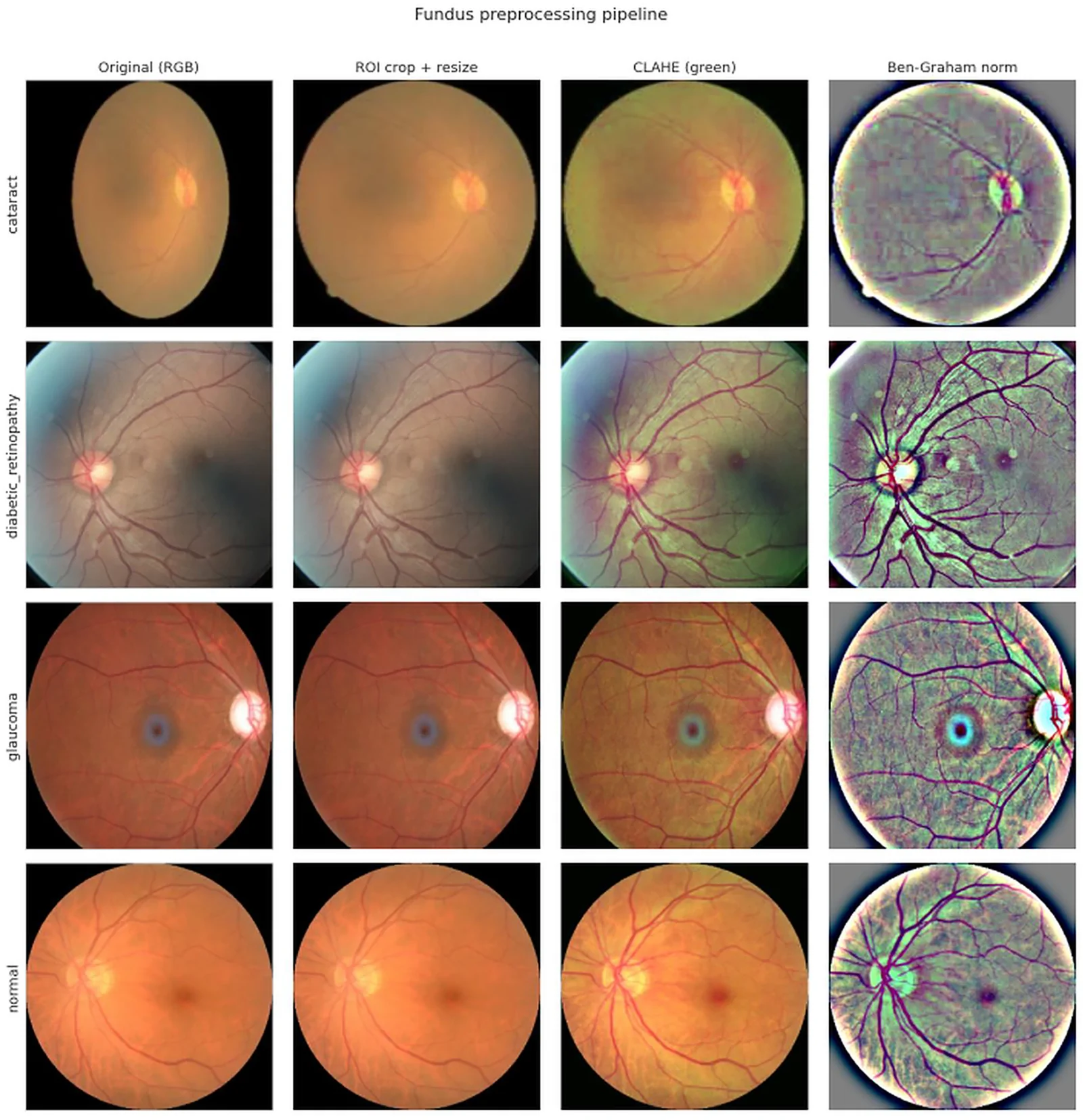
Fundus preprocessing pipeline for one representative image per class: original RGB image, region-of-interest crop and resize, contrast-limited adaptive histogram equalisation on the green channel, and Ben-Graham illumination normalisation.

### 3.3 Model parameters and complexity

QICAF-Net comprises a Swin Transformer global branch and an EfficientNet local branch, each initialised from ImageNet-pretrained weights, whose token representations are projected to a common 256-dimensional latent space and fused by the QICAF module before a compact classification head. The principal architectural hyperparameters are summarised in **Table 2**. The dual-branch design is intentionally heavier than a single backbone; this cost is quantified and discussed in the comparative analysis, where it is weighed against the interpretability the architecture provides.

**Table 2:** Principal architectural and training hyperparameters of QICAF-Net.

Component	Configuration
Global branch	Swin Transformer (small variant), ImageNet-pretrained, token output
Local branch	EfficientNet (B4 variant), ImageNet-pretrained, spatial feature map
Projection dimension	256 (shared latent space for both branches)
QICAF attention heads	4
Input resolution	288 x 288 x 3
Classifier head	Linear (256 to 128), GELU, dropout 0.3, linear (128 to 4)
Optimiser	AdamW, differential learning rates for backbones and new modules
Objective	Class-weighted focal loss (gamma = 2) with label smoothing 0.1
Regularisation	Exponential moving average, mixup, anatomy-preserving augmentation
Schedule	Cosine decay with linear warm-up; gradient accumulation

### 3.4 Implementation and hardware

The framework was implemented in PyTorch with the timm library providing the pretrained backbones. All experiments were executed on a single NVIDIA T4 graphics processing unit within the Kaggle notebook environment. Mixed-precision computation and gradient accumulation were used to accommodate the dual-branch model at the chosen input resolution within the available memory. To ensure reproducibility, random seeds were fixed for all stochastic components, and the data partitioning was performed with a fixed random state so that the same folds and held-out test set were used consistently across the internal experiments. Partitioning was performed at the image level with stratification by class, because the public dataset provides no patient or source identifiers; consequently patient-level and source-level splitting could not be applied, and the possibility of same-patient or same-source leakage cannot be fully excluded (discussed in Section 8).

### 3.5 Evaluation metrics

Given the multi-class setting, performance was quantified using accuracy and the macro-averaged precision, recall and F1-score, so that every class contributes equally regardless of residual imbalance. Let TP_c, TN_c, FP_c and FN_c denote the true positives, true negatives, false positives and false negatives for class c, and let K = 4 be the number of classes. Accuracy is the proportion of correctly classified images across all classes, defined in Equation 1.

(1) Accuracy=(Σ_c TP_c)/N,N=total number of images

Precision for a class is the proportion of images predicted as that class that are correct, and recall is the proportion of images truly belonging to that class that are recovered, given in Equations 2 and 3. Both are then macro-averaged over the K classes.

(2) Precision_c=TP_c/(TP_c+FP_c)

(3) Recall_c=TP_c/(TP_c+FN_c)

The F1-score is the harmonic mean of precision and recall for each class, Equation 4, and the macro F1-score, Equation 5, is the unweighted mean of the per-class F1-scores. The macro F1-score is treated as the primary metric because it jointly reflects precision and recall across all four categories and is not dominated by any single class.

(4) F1_c=2·(Precision_c·Recall_c)/(Precision_c+Recall_c)

(5) Macro-F1=(1/K)·Σ_c F1_c

Cohen’s kappa, Equation 6, measures agreement between predictions and ground truth corrected for the agreement expected by chance, where p_o is the observed agreement and p_e is the expected chance agreement; a value approaching one indicates strong agreement beyond chance.

(6) κ=(p_o−p_e)/(1−p_e)

In addition, the one-versus-rest area under the receiver operating characteristic curve is reported as a threshold-independent measure of class separability, computed per class and macro-averaged. For cross-validation, all metrics are summarised as the mean and sample standard deviation across folds. Statistical comparisons between models use paired tests across matched evaluation units; the primary comparison between QICAF-Net and the matched concatenation model uses three matched seeds, with the exact values reported in Section 5.6. The numbering of equations continues into the methodology, where Equations 7–20 formalise the model.

## 4 Proposed methodology

QICAF-Net is a dual-branch classifier whose novelty resides in the fusion stage. A global branch and a local branch independently transform the preprocessed fundus image into token representations; these are projected into a shared latent space and combined by the Quantum-Inspired Cross-Attention Fusion module, whose output is passed to a classification head and, in parallel, to the explainability stage. The complete end-to-end pipeline is shown in **Figure 3**, the internal structure of the fusion module is expanded in **Figure 4**, and the quantum-inspired operations are additionally rendered in circuit-diagram notation in **Figure 5**. This section formalises each stage.

**Figure 3:**
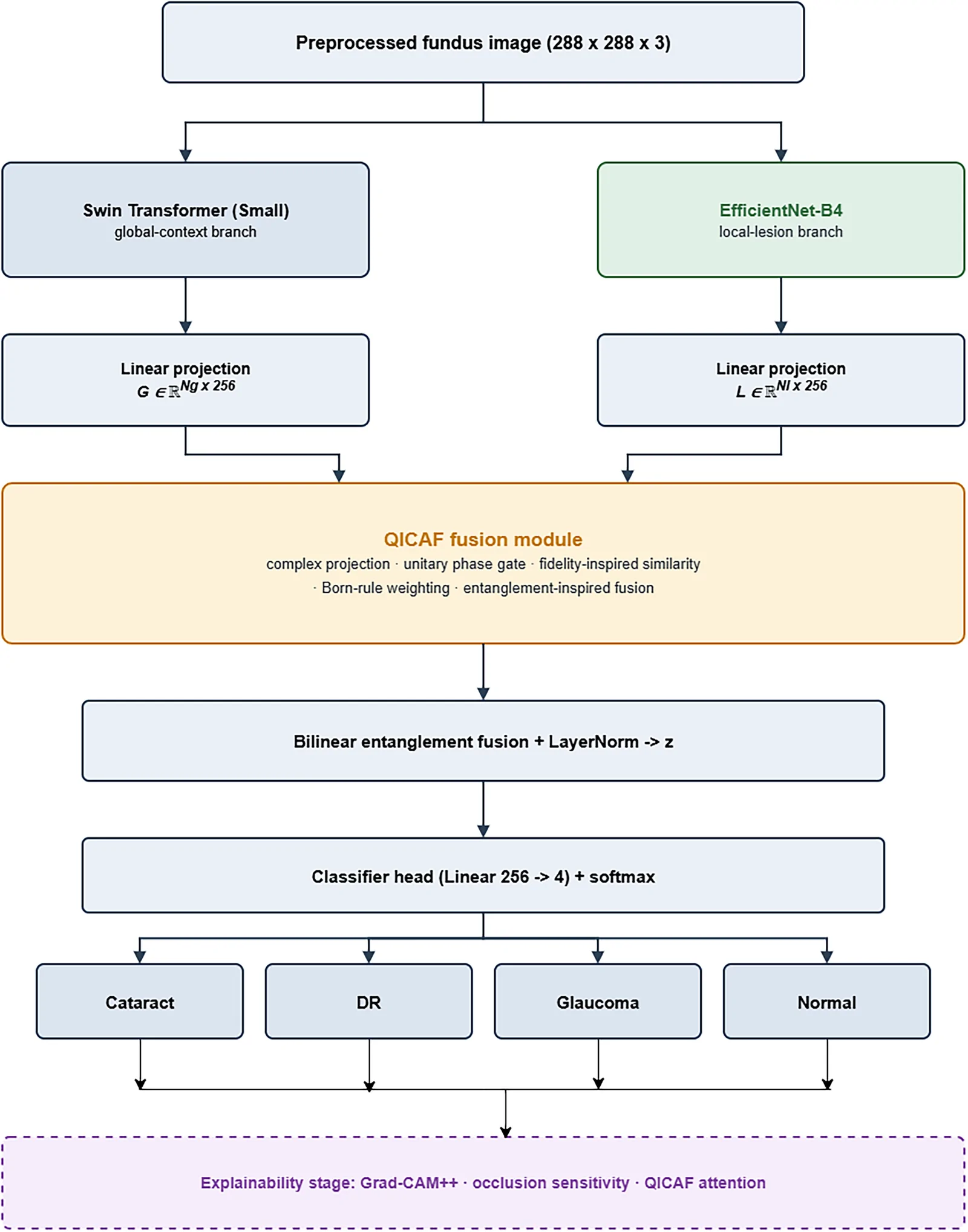
Overall architecture of QICAF-Net. The preprocessed fundus image is passed to a global transformer branch and a local convolutional branch; both are projected to a shared 256-dimensional space and combined by the QICAF module through complex projection, a unitary gate, fidelity similarity, Born-rule weighting and entanglement fusion, followed by the classifier head and the explainability stage.

**Figure 4:**
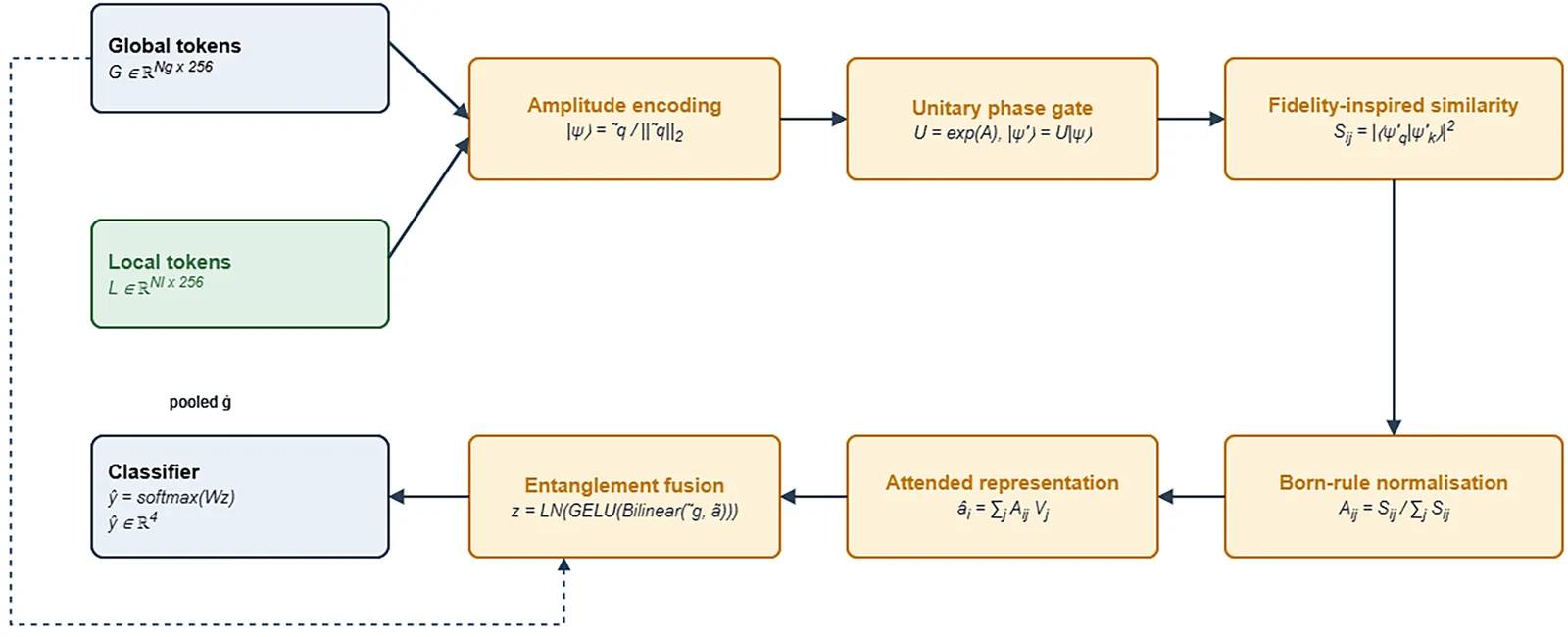
Expanded view of the QICAF fusion pathway. The global and local token representations are amplitude-encoded, transformed by a unitary phase gate, compared through a fidelity-inspired similarity with Born-rule normalisation, and combined by an entanglement-inspired bilinear fusion before classification.

**Figure 5:**
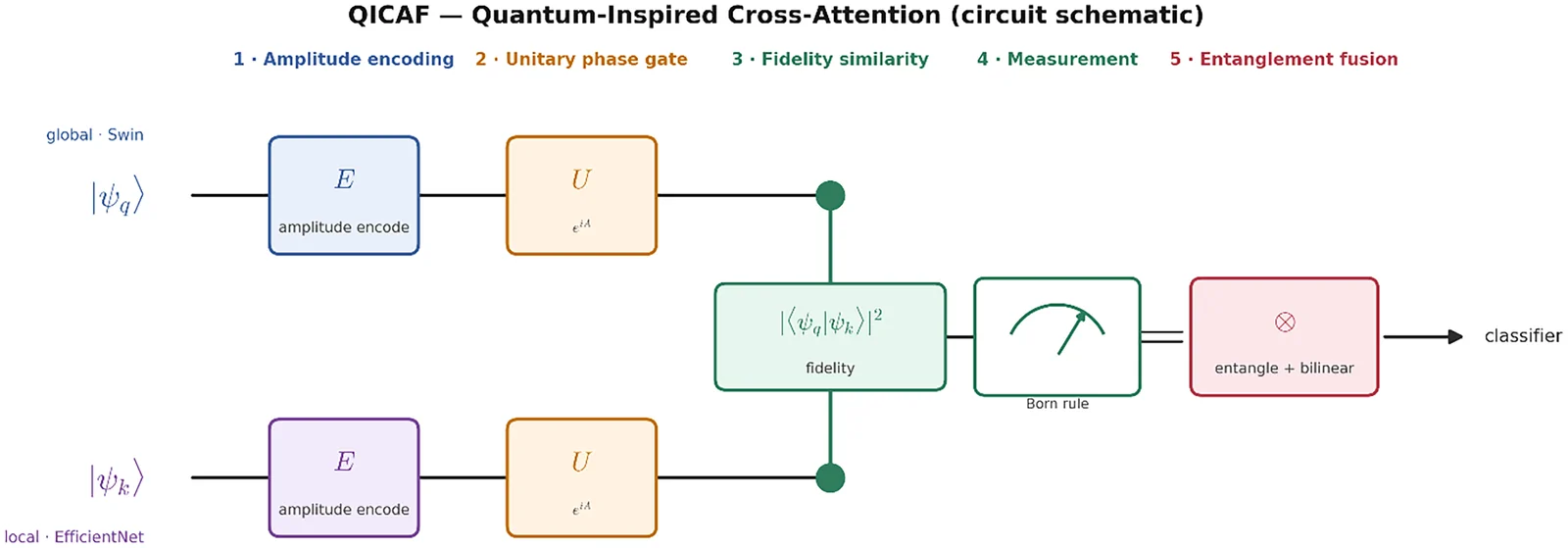
Schematic of the QICAF module in quantum-circuit notation. The two state wires carry amplitude-encoded global and local tokens through a unitary phase gate, a fidelity-inspired coupling, a Born-rule measurement and an entanglement-inspired fusion. All operations are realised as classical linear algebra; the image is a notation-level schematic and does not represent a physical quantum circuit.

### 4.1 Dual-branch feature extraction

Let X denote the preprocessed input image. The global branch g and the local branch l produce token sequences that are linearly projected to a common dimension. The global branch yields a set of tokens capturing long-range structure, and the local branch yields a set of tokens capturing focal lesion texture, as expressed in Equations 7, 8, where W_g and W_l are learnable projection matrices and d is the shared latent dimension.

(7) G=W_g·SwinTokens(X), G∈ℝ^{N_g×d}

(8) L=W_l·EffNetTokens(X), L∈ℝ^{N_l×d}

Here N_g and N_l are the numbers of global and local tokens respectively, and both branches are initialised from ImageNet-pretrained weights and fine-tuned end to end. The design assigns complementary roles to the two branches. The transformer branch, through its windowed self-attention, encodes long-range spatial relationships such as the geometry of the optic disc and the overall course of the retinal vasculature, which are the discriminative cues for glaucoma and for the global assessment of a healthy fundus. The convolutional branch, through its hierarchical receptive fields, preserves fine local texture such as microaneurysms, exudates and haemorrhages, which are the discriminative cues for diabetic retinopathy, and the diffuse opacity characteristic of cataract. Projecting both token sets to a common dimension d places the two heterogeneous representations in a shared space so that they can be compared and combined. In the cross-attention that follows, the global tokens act as the query source and the local tokens act as the key and value source, so that the global structural context actively interrogates the local lesion evidence rather than the two branches being merged by an unweighted operation. This asymmetric assignment reflects the clinical reasoning that structural context guides where fine lesion evidence should be weighted most heavily.

### 4.2 Amplitude encoding

The quantum-inspired treatment begins by representing each token as a quantum state. A complex-valued projection maps a real token to a complex vector, and the vector is normalised to unit length so that it lies on the unit hypersphere, the classical analogue of a normalised quantum state. For a query token, the complex state is formed and normalised as in Equations 9 and 10, where the subscripts re and im denote the learnable real and imaginary projections and the analogous construction applies to the key tokens.

(9) q˜=W_q^{re} q+i·W_q^{im} q

(10) $$|\psi \_q\rangle =\tilde{q}/\left\|\tilde{q}\right\|\_2$$

The motivation for this construction is that a normalised quantum state is a unit vector in a complex Hilbert space, and the amplitude-encoding step maps each real token onto exactly such an object. The learnable real and imaginary projections W_q^{re} and W_q^{im} allow the network to place each token at a data-dependent position and phase on the unit hypersphere, so the encoding is not a fixed transformation but is optimised jointly with the rest of the model. Two properties follow that are exploited later. First, because every encoded token has unit norm, the inner product between two encoded tokens is bounded and directly interpretable as an overlap between states. Second, the complex representation carries phase information that a purely real projection cannot, and it is this phase that the unitary gate of the next stage manipulates. Normalisation is therefore not cosmetic: it is the condition that makes the subsequent fidelity and Born-rule measurement operations well defined. The identical amplitude encoding, with its own learnable projections, is applied to the key tokens derived from the local branch to obtain the key states, so that queries and keys inhabit the same encoded space.

### 4.3 Unitary phase gate

Each encoded state is then evolved by a learnable unitary transformation, the classical counterpart of quantum-gate evolution. A unitary operator is guaranteed by exponentiating a skew-Hermitian matrix, as in Equations 11, 12, where A is parameterised from learnable weights so that A^H = -A, and matrix exponentiation yields a unitary U with U U^H = I.

(11) A=i·H, H=H^H(Hermitian)⇒A^H=−A

(12) $$U=exp(A),|\psi^{\prime }\rangle =U|\psi \rangle ,\left\||\psi^{\prime }\rangle \right\|=\left\||\psi \rangle \right\|$$

The construction guarantees unitarity by design. Any matrix of the form U = exp(A) with A skew-Hermitian is unitary, and skew-Hermiticity is enforced by building A from a learnable Hermitian generator multiplied by the imaginary unit, as in Equation 11. The matrix exponential is differentiable, so the generator is trained by ordinary back-propagation together with the rest of the network. Because a unitary transformation preserves inner products and therefore norms, the evolved state in Equation 12 remains a valid unit-norm quantum-state analogue; the gate rotates the state within the Hilbert space without distorting its magnitude. Conceptually, this is the classical counterpart of applying a quantum gate to a qubit register: the information content is re-expressed in a learned basis while the probabilistic normalisation is conserved. The phase rotation introduced by the gate is the genuinely quantum-inspired degree of freedom of the module, since it has no direct equivalent in real-valued dot-product attention, and separate gates with independent generators are learned for the query and key states so that the two can be aligned in a task-adaptive manner. A numerical check confirmed that the learned operator satisfies the unitarity condition to within a tolerance of 10^-7.

### 4.4 Fidelity-inspired similarity and born-rule normalisation

Classical scaled dot-product attention scores query-key pairs by an inner product. In QICAF this is replaced by a fidelity-inspired similarity: the squared magnitude of the complex inner product between the evolved query and key states, given in Equation 13. This quantity is the classical analogue of the quantum state fidelity, or Born-rule transition probability, between the two states.

(13) $$S\_\left\{ij\right\}={\left|\langle \psi^{\prime }\_\left\{q,i\right\}|\psi^{\prime }\_\left\{k,j\right\}\rangle \right|}^{2}$$

The complex inner product is computed from the real and imaginary components as in Equation 14, and its squared magnitude in Equation 15, where the superscripts denote the real and imaginary parts of the evolved states.

(14) $$\langle \psi^{\prime }\_q|\psi^{\prime }\_k\rangle =\left(q\_r\cdot k\_r+q\_i\cdot k\_i\right)+i\cdot \left(q\_r\cdot k\_i-q\_i\cdot k\_r\right)$$

(15) $$S\_\left\{ij\right\}={\left(q\_r\cdot k\_r+q\_i\cdot k\_i\right)}^{2}+{\left(q\_r\cdot k\_i-q\_i\cdot k\_r\right)}^{2}$$

The similarity scores are non-negative by construction. They are converted to attention weights by a Born-rule normalisation, that is, by dividing each score by the sum of scores over all keys, as in Equation 16. This gives the attention weights the interpretation of measurement probabilities, in contrast to the exponential softmax of classical attention.

(16) A_{ij}=S_{ij}/Σ_j S_{ij}, Σ_j A_{ij}=1

The attended representation for each query token is the weighted combination of value tokens, Equation 17, where V is the value projection of the local tokens. The multi-head form applies Equations 9–17 independently per head and concatenates the results.

(17) $$\hat{a}\_i=\Sigma \_j\;A\_\left\{ij\right\}\cdot V\_j$$

A numerical verification confirmed that the learned operator satisfies U U^H = I to within 10^-7, that the gate preserves state norm to within 10^-7, and that the normalised attention weights form a valid probability distribution summing to one. Because the unit-norm constraint is applied before the multi-head split, the raw fidelity is not strictly bounded above by one; the term fidelity-inspired similarity is therefore used throughout, and the Born-rule normalisation renders the resulting weights well defined regardless.

### 4.5 Entanglement-inspired fusion and classification

The final stage combines the pooled global representation with the pooled attended representation through an entanglement-inspired bilinear interaction, the classical analogue of forming a correlated joint state, as in Equation 18, where g-bar and a-bar are the mean-pooled global and attended representations and the bilinear form couples them multiplicatively.

(18) $$z=LayerNorm\left(GELU\left(Bilinear\left(\bar{g},\bar{a}\right)\right)\right)$$

The rationale for a bilinear interaction is that a simple sum or concatenation of the global and attended representations models only additive, first-order relationships between them, whereas a bilinear form captures multiplicative, second-order interactions in which the contribution of each global feature is modulated by the attended local features and vice versa. This is the classical analogue of forming a correlated joint state from two subsystems, in which the combined description is not separable into an independent product of its parts. In practice the interaction is followed by layer normalisation and a non-linear activation to stabilise the scale of the fused representation and to introduce the non-linearity required for expressive classification. The fused vector z is then passed to the classification head, and class probabilities are produced by a softmax over the four categories, Equation 19, where W_c and b_c are the head parameters. It is reported transparently, and analysed in Section 5, that although this entanglement-inspired stage is well motivated, its measured contribution to classification accuracy over simpler fusion is small; its role is therefore best understood as part of the interpretable, physically-motivated formulation rather than as a decisive source of accuracy.

(19) $$\hat{y}=softmax\left(W\_c\cdot z+b\_c\right),\;\hat{y}\in {\mathbb{R}}^{4}$$

### 4.6 Training objective

The network is optimised with a class-weighted focal loss computed in full precision for numerical stability, given in Equation 20, where p_c is the predicted probability of the target class after label smoothing, alpha_c is the per-class weight that mildly up-weights the harder glaucoma category, and gamma is the focusing parameter. Label smoothing distributes a small probability mass across non-target classes to improve calibration.

(20) L=−∑_c α_c(1−p_c)^γ·y_c·log(p_c)

A cosine learning-rate schedule with linear warm-up, exponential moving averaging (EMA) of the weights, mixup augmentation and gradient accumulation are used during optimisation. At inference, six-view test-time augmentation is applied and the predicted probabilities are averaged to produce the final decision.

### 4.7 Quantum-inspired circuit view and overall algorithm

**Figure 5** renders the fusion module in the notation of a quantum circuit to make the correspondence between each operation and its quantum analogue explicit. Two state wires carry the amplitude-encoded global and local tokens; state-preparation gates perform amplitude encoding; unitary gates apply the learned phase rotation; a two-wire coupling computes the fidelity-inspired similarity; a measurement symbol denotes the Born-rule normalisation; and an entanglement gate performs the bilinear fusion. It must be emphasised again that the circuit is a schematic in quantum notation and that every operation is executed as classical linear algebra; the figure carries this statement to prevent any misreading as physical quantum computation.

The complete training and inference procedure of QICAF-Net, including the QICAF fusion, theclass-weighted focal objective, exponential moving averaging and six-view test-time augmentation, isgiven in **Algorithm 1**. The algorithm makes explicit the order in which the quantum-inspired operations are applied within each attention head and how the trained model is deployed at inference.

Algorithm 1 **QICAF-Net: training and inference.**

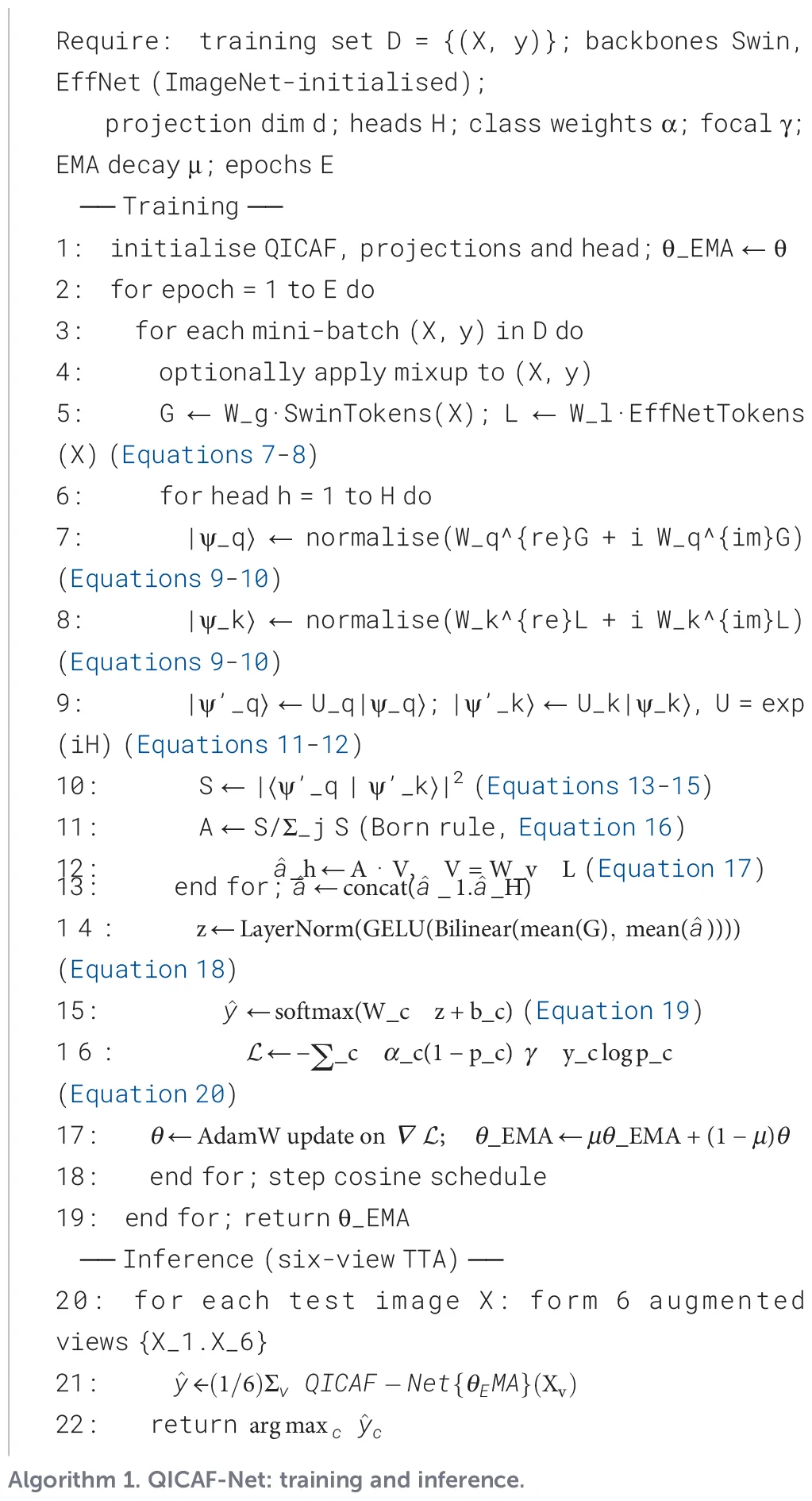

## 5 Results and discussion

This section reports the internal cross-validation and test performance, the training behaviour, the confusion structure and separability, the baseline comparison, the component ablation, the paired significance analysis, the external validation and the explainability analysis. Throughout, the primary internal metric is the macro F1-score, and results that require careful interpretation are discussed openly.

### 5.1 Training behaviour

**Figure 6** shows the loss, accuracy and macro-F1 curves for the single-split training run. The validation loss decreases smoothly and plateaus, and the validation accuracy and macro-F1 rise to approximately 0.95 before stabilising. A characteristic and expected feature of these curves is that the training-time accuracy is lower than the validation accuracy. This is not a sign of underfitting or of a data problem; it is a direct consequence of the training-time regularisation. Mixup blends pairs of images and their labels, so a substantial fraction of training batches contain deliberately ambiguous images whose single-label accuracy is mechanically penalised, and the heavy augmentation makes training images harder than the clean validation images. In addition, the exponential moving average weights used for validation generalise slightly better than the raw weights at any given step. The gap therefore reflects an intentionally harder training distribution rather than a defect, and training-time accuracy is consequently not treated as a headline metric.

**Figure 6:**
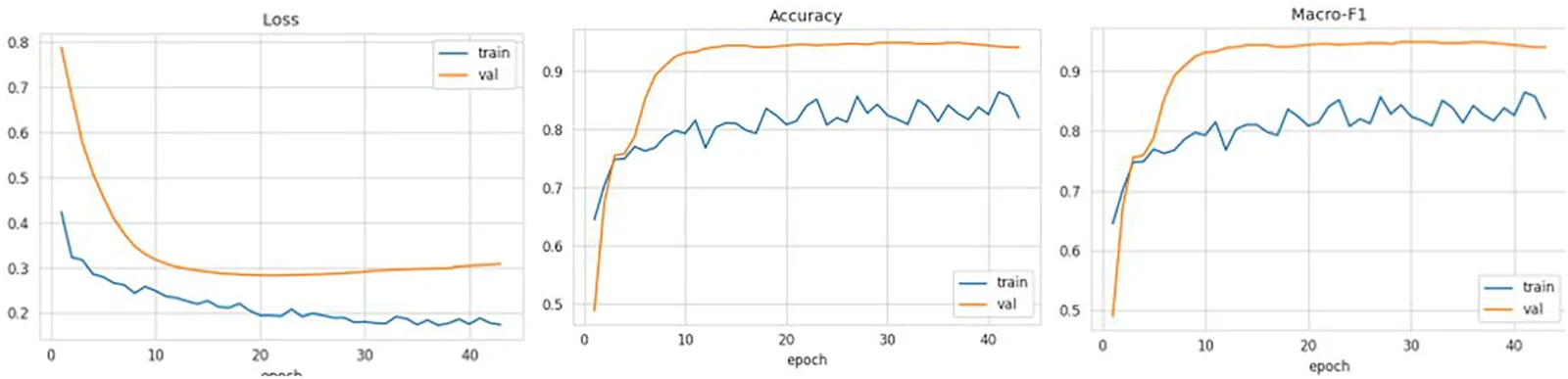
Training and validation curves for the single-split run: loss (left), accuracy (centre) and macro-F1 (right). Validation exceeds training accuracy because mixup and strong augmentation make the training batches deliberately harder than the clean validation set.

### 5.2 Internal classification performance

On the held-out internal test partition, the representative QICAF-Net model with six-view TTA achieved an accuracy of 0.9384, a macro-F1 of 0.9371 and a macro one-versus-rest AUC of 0.9947. This single representative model, trained to 45 epochs, is the fixed instance used for all per-class, calibration, explainability and external analyses; the five-fold and multi-seed summaries are reported in Sections 5.3 and 5.5. The per-class results in **Table 3** show near-perfect diabetic retinopathy detection and strong cataract performance, with the residual error concentrated in the glaucoma-versus-normal distinction, a well-known difficulty because early glaucomatous change can closely resemble a healthy fundus. The class-weighted objective mildly favouring glaucoma was introduced specifically to mitigate this effect.

**Table 3:** Per-class performance of QICAF-Net on the internal held-out test partition.

Class	Precision	Recall	F1-score	Support
Cataract	0.961	0.955	0.958	156
Diabetic retinopathy	1.000	1.000	1.000	165
Glaucoma	0.934	0.841	0.885	151
Normal	0.864	0.950	0.905	161
**Macro average**	**0.940**	**0.937**	**0.937**	**633**

Bold values indicate the macro-averaged performance across the four classes.

The confusion matrices in **Figure 7** confirm this structure. Diabetic retinopathy is classified without error, cataract is recovered with high fidelity, and the principal off-diagonal mass corresponds to a small number of glaucoma cases predicted as normal and vice versa. The one-versus-rest receiver operating characteristic curves in **Figure 8** show excellent separability for every class, with areas under the curve between 0.991 and 1.000, indicating that the ranking of predictions is highly reliable even where the hard-decision boundary between glaucoma and normal introduces a small number of errors.

**Figure 7:**
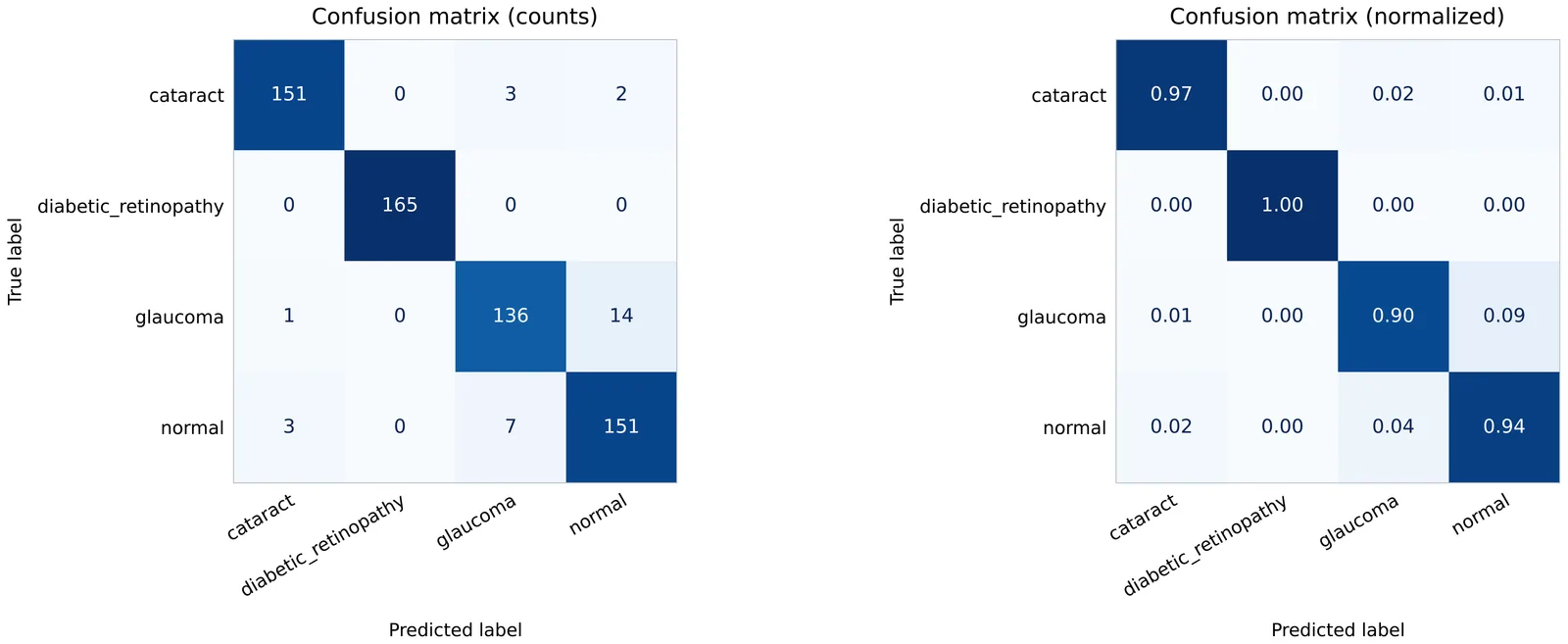
Confusion matrices on the internal test partition as raw counts (left) and row-normalised proportions (right). Diabetic retinopathy is classified without error; residual confusion is concentrated between glaucoma and normal (per-class counts correspond to the updated **Table 3**).

**Figure 8:**
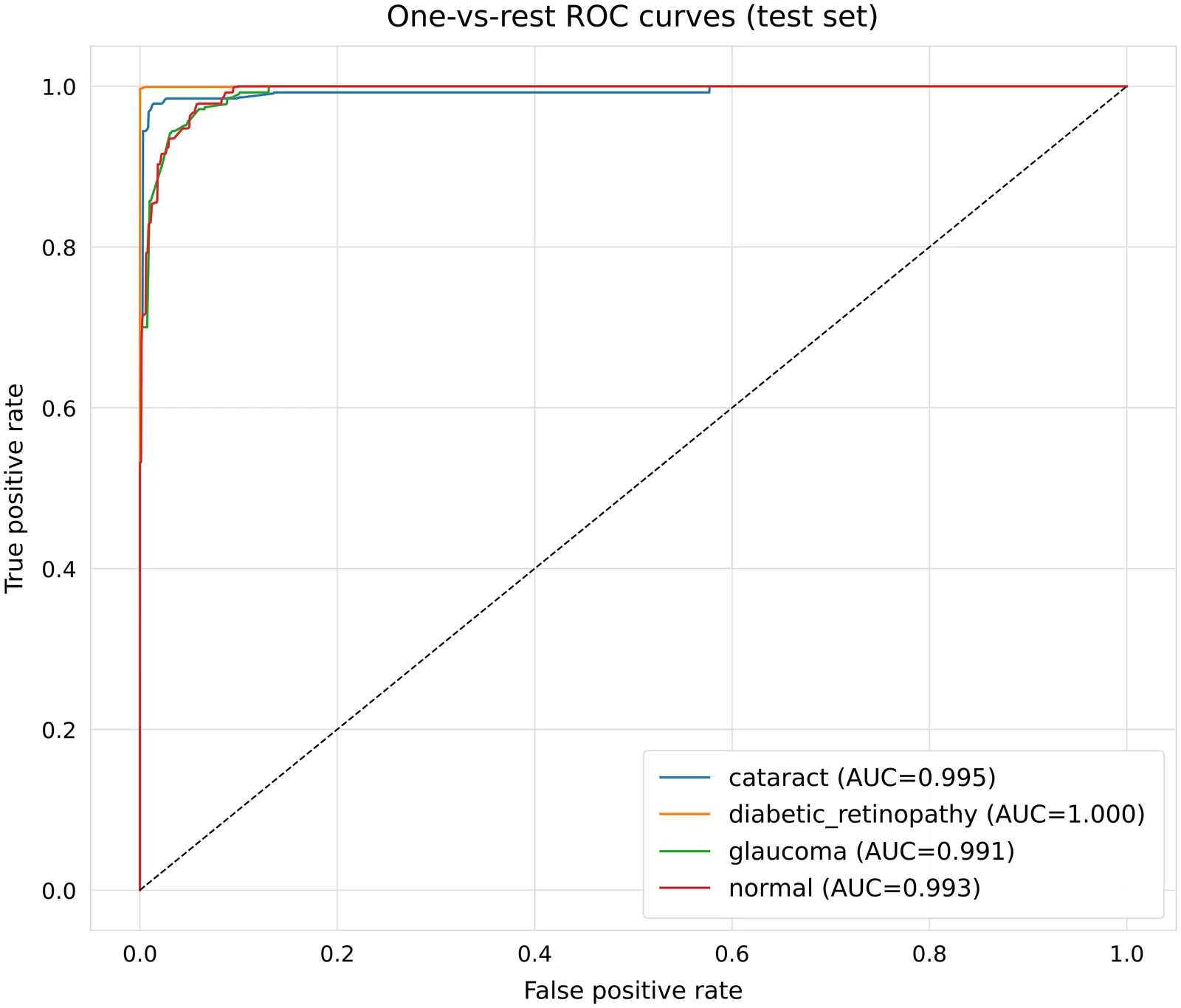
One-versus-rest receiver operating characteristic curves on the internal test partition, with areas under the curve between 0.991 and 1.000 across the four classes.

### 5.3 Cross-validation robustness

To assess stability beyond a single partition, stratified five-fold cross-validation was performed (45 epochs per fold). The per-fold macro-F1 values under six-view TTA were 0.9542, 0.9293, 0.9388, 0.9263 and 0.9302, giving a mean macro-F1 of 0.9358 (sample SD 0.0113; 95% CI 0.9218–0.9498), as visualised in **Figure 9**; accuracy was 0.9367 (95% CI 0.9228–0.9506) and macro-AUC 0.9914 (95% CI 0.9885–0.9943). A complementary three-seed evaluation of the full model (seeds 42, 123, 3407) gave a macro-F1 of 0.9420 ± 0.0071, consistent with the cross-validation estimate. The narrow spread of approximately three percentage points between the best and worst folds indicates that the model is stable and does not depend on a fortuitous split. The cross-validation mean is regarded as the headline robustness figure for the internal evaluation, while the single-split test result is reported as the performance of one trained instance. Fold-level 95% confidence intervals were computed from the five fold values using the Student’s t distribution with four degrees of freedom, and external confidence intervals (Section 5.7) by 2,000 image-level bootstrap resamples. The internal effect of test-time augmentation is isolated in Section 5.15 (**Table 4**).

**Figure 9:**
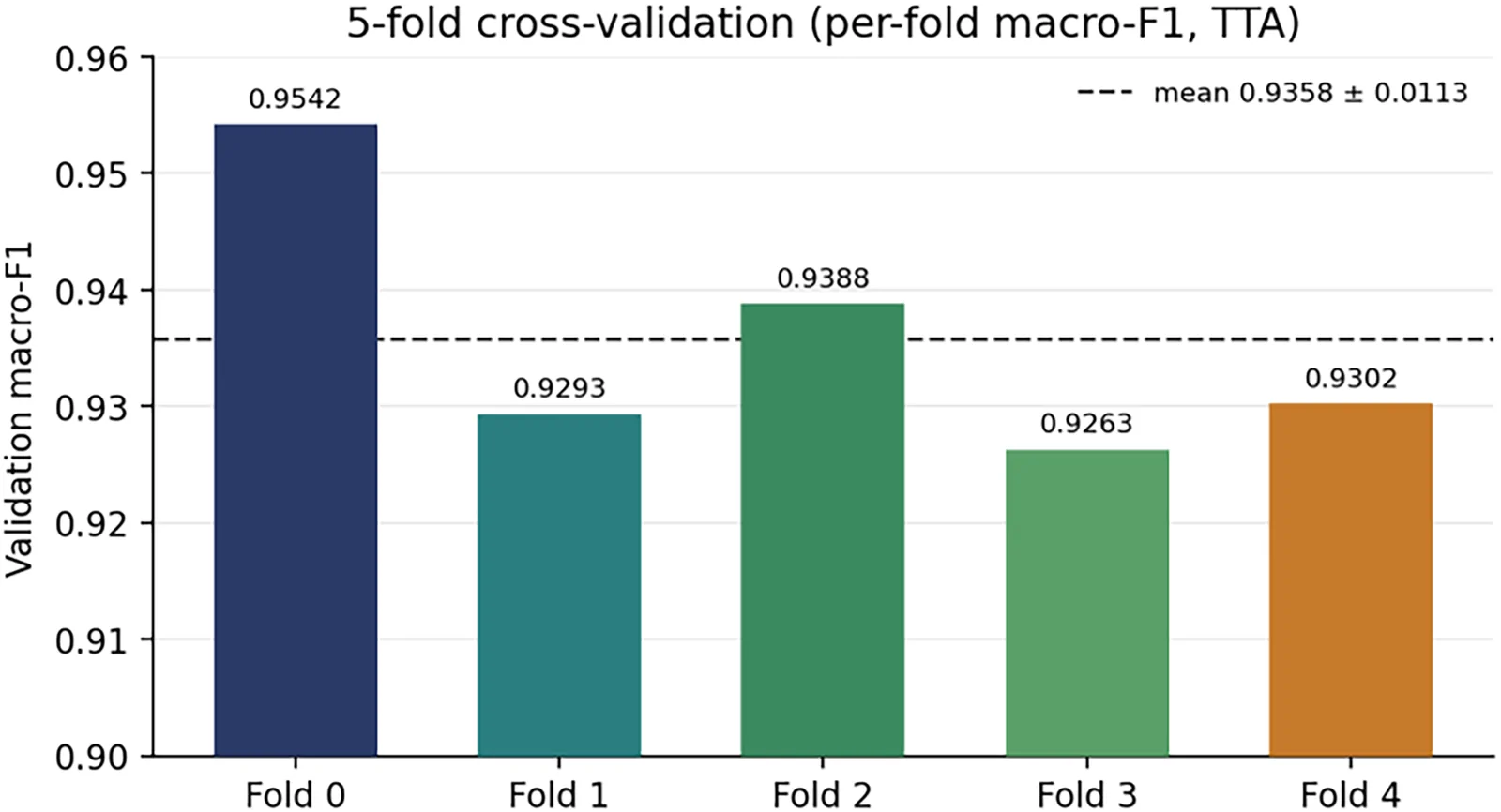
Per-fold validation macro-F1 under stratified five-fold cross-validation. The dashed line marks the mean of 0.9358 with a standard deviation of 0.0113.

**Table 4:** Internal five-fold cross-validation with and without six-view test-time augmentation, isolating the TTA contribution (mean ± SD across folds).

Internal condition (5-fold CV)	Accuracy	Macro-F1	Macro-AUC
No TTA	0.9329 ± 0.0128	0.9319 ± 0.0130	0.9894 ± 0.0044
Six-view TTA	0.9367 ± 0.0112	0.9358 ± 0.0113	0.9914 ± 0.0023
**Δ (TTA − no TTA)**	**+0.0038**	**+0.0039**	**+0.0020**

Bold values indicate the difference attributable to six-view test-time augmentation.

### 5.4 Comparison with baseline models

QICAF-Net was compared against five standard single-backbone classifiers, ResNet-50, Swin-Tiny, ViT-Small, EfficientNet-B3 and DenseNet-121, trained and evaluated under the identical preprocessing and six-view TTA protocol so that only the architecture differed. The results are shown in **Figure 10** and summarised in **Table 5**. Under matched TTA, QICAF-Net (macro-F1 0.9420) is competitive but does not achieve the highest macro-F1: Swin-Tiny (0.9583) and ViT-Small (0.9487) exceed it. This is reported transparently and is consistent with the central thesis of the study — the value of the dual-branch design is best understood not as a decisive accuracy advantage over every backbone but as competitive accuracy combined with the interpretable cross-modal attention that a single backbone does not provide. TTA contributed a positive gain for the convolutional baselines (e.g. +0.0225 for EfficientNet-B3) but a marginal change for the transformer baselines, underlining that TTA effects are architecture-dependent.

**Figure 10:**
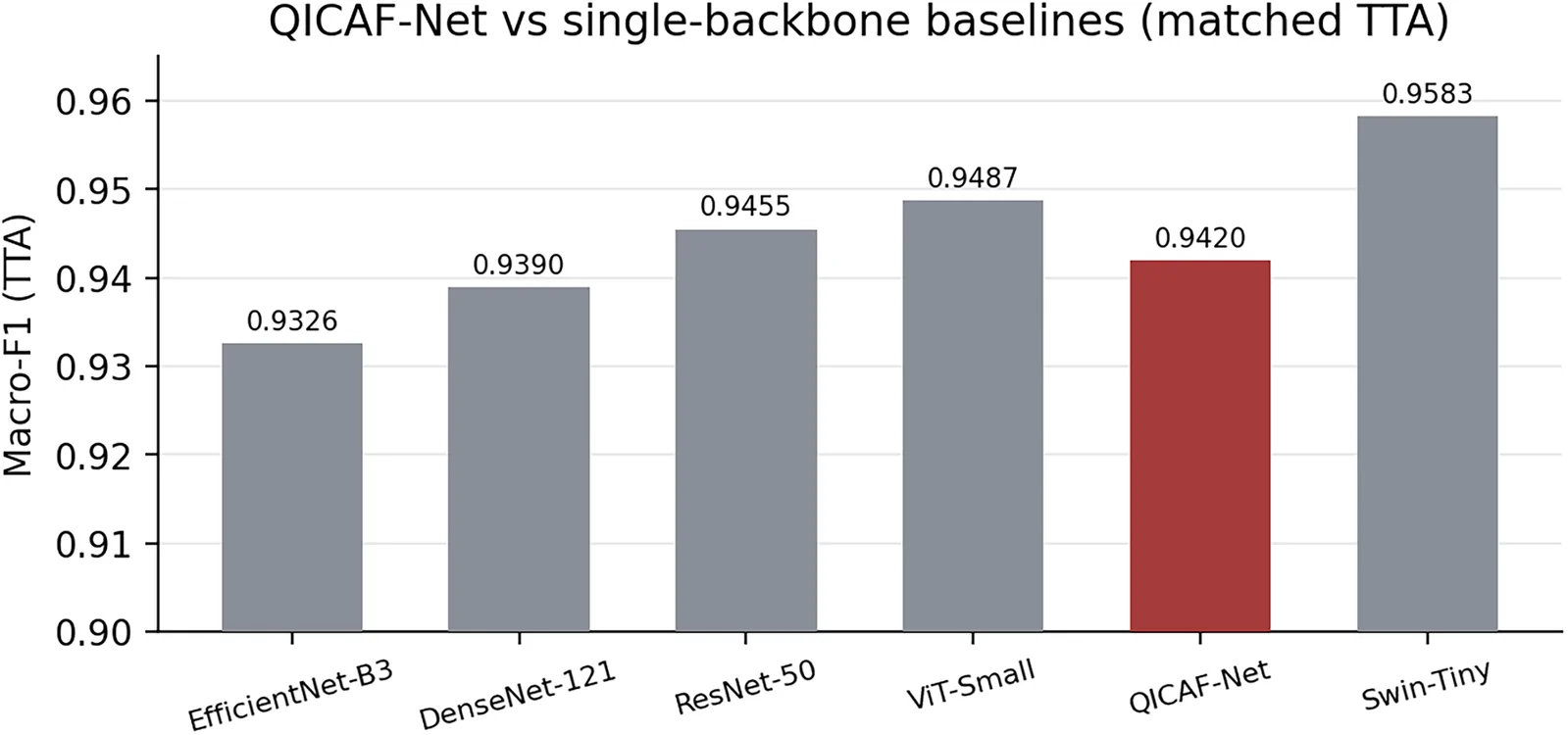
Comparison of QICAF-Net with single-backbone baseline models. Under matched six-view TTA, QICAF-Net is competitive with but does not exceed the strongest transformer backbones (Swin-Tiny, ViT-Small).

**Table 5:** Comparison of QICAF-Net with single-backbone baselines under a matched preprocessing and six-view TTA protocol (macro-F1); QICAF-Net is the three-seed mean and baselines are single-split.

Model	Type	Macro-F1 (TTA)
ResNet-50	Convolutional	0.9455
Swin-Tiny	Transformer	0.9583
ViT-Small	Transformer	0.9487
EfficientNet-B3	Convolutional	0.9326
DenseNet-121	Convolutional	0.9390
**QICAF-Net (proposed)**	**Dual-branch fusion**	**0.9420 ± 0.0071**

Bold values indicate the proposed model.

### 5.5 Cumulative ablation of the QICAF components

To isolate the contribution of each quantum-inspired component and to provide multi-seed evidence for each component, the six-configuration cumulative ablation was repeated over three independent seeds (42, 123, 3407), giving 18 training runs (20 epochs each). The three-seed mean macro-F1 values were 0.9414 ± 0.0046 for concatenation, 0.9426 ± 0.0077 for classical cross-attention, 0.9426 ± 0.0038 with the complex projection, 0.9403 ± 0.0032 with the unitary gate, 0.9419 ± 0.0033 with fidelity and Born-rule normalisation, and 0.9420 ± 0.0071 for the full module, as shown in **Figure 11**, **Table 6**. Two observations follow. First, the quantum-inspired configurations all lie within a narrow band around the concatenation baseline, and the differences between them are of the same order as the fold-to-fold variation observed in cross-validation; the evidence therefore supports accuracy parity across fusion strategies rather than a decisive advantage for any single component. Second, the ablation used a reduced 20-epoch budget to isolate relative component effects; this differs from the 45-epoch budget of the headline model and five-fold evaluation and is disclosed rather than smoothed over.

**Figure 11:**
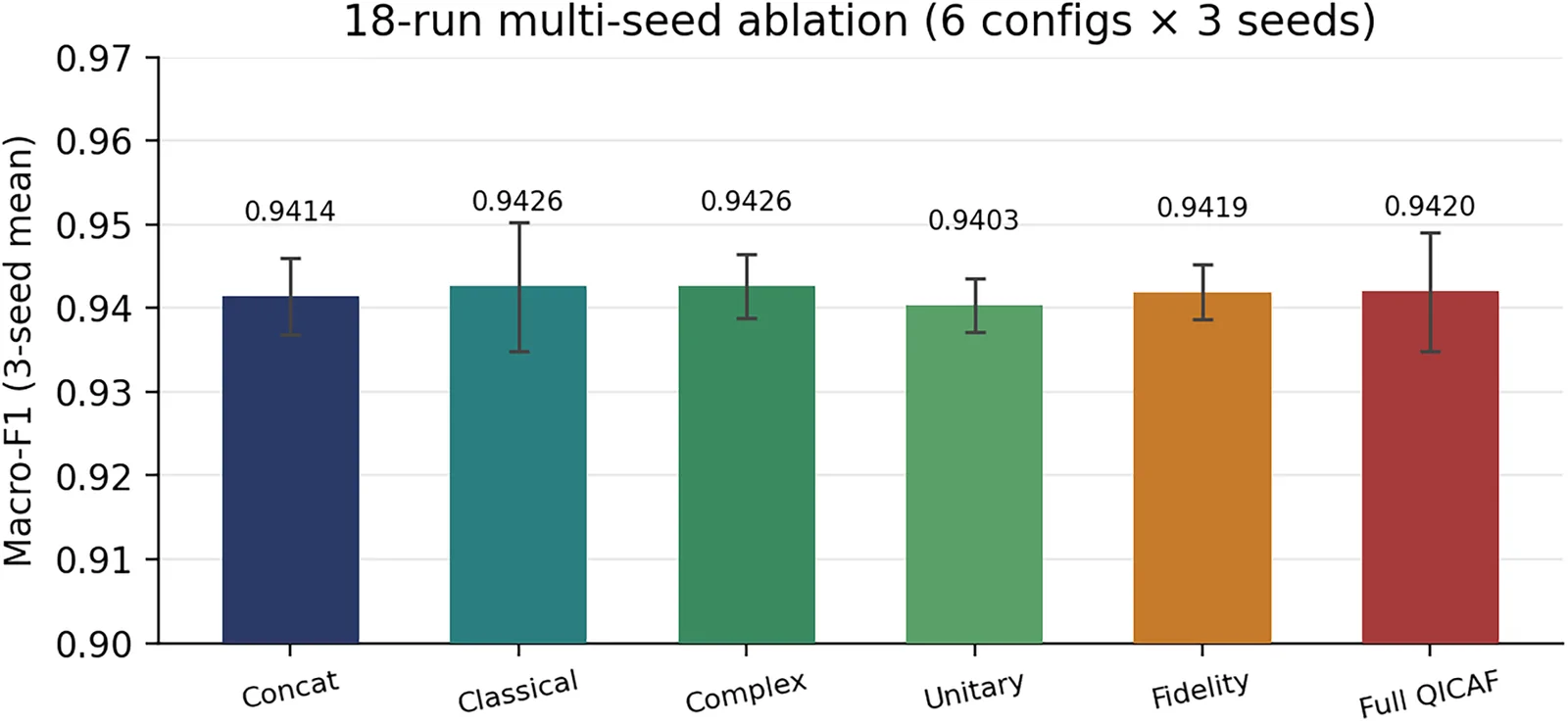
Cumulative ablation of the QICAF components. Three-seed means with standard-deviation bars; all configurations lie within a narrow band around the concatenation baseline, with differences comparable to between-seed variation.

**Table 6:** Cumulative ablation of QICAF components (three-seed mean macro-F1, 20 epochs).

Configuration	Added component	Macro-F1 (3-seed mean)
Concatenation	baseline	0.9414 ± 0.0046
Classical cross-attention	softmax attention	0.9426 ± 0.0077
+ Complex projection	amplitude encoding	0.9426 ± 0.0038
+ Unitary phase gate	unitary evolution	0.9403 ± 0.0032
+ Fidelity and Born rule	measurement	0.9419 ± 0.0033
**Full QICAF (+ entanglement)**	**bilinear fusion**	**0.9420 ± 0.0071**

Each configuration adds one component to the previous one.

Bold values indicate the full proposed QICAF configuration.

### 5.6 Paired significance analysis

Because the ablation differences were small, a paired three-seed significance test was conducted between the full QICAF model and a matched dual-branch model in which the QICAF module was replaced by mean-pooling and concatenation, using the three matched seeds (42, 123, 3407) of the multi-seed ablation. This comparison isolates the contribution of the quantum-inspired attention itself. The per-seed values are given in **Table 7**. Full QICAF achieved a mean macro-F1 of 0.9420 and the matched concatenation model achieved 0.9414, a mean paired difference of +0.0006. A paired t-test returned p = 0.887 and a Wilcoxon signed-rank test returned p = 1.000, so the difference is not statistically significant; because only three matched observations are available, the test has low statistical power and is presented as supporting evidence of parity rather than a definitive demonstration. The honest interpretation is that the quantum-inspired attention matches, but does not exceed, classical concatenation in classification accuracy on this dataset. The contribution of the module is therefore its novel and interpretable formulation, and the interpretability it enables, rather than a raw accuracy improvement. Reporting this null result transparently is a deliberate strength of the study, since a claim of superiority would not withstand an independent re-run of the concatenation baseline.

**Table 7:** Paired three-seed comparison between full QICAF and a matched dual-branch concatenation model (macro-F1).

Seed	Concatenation macro-F1	Full QICAF macro-F1	Difference
42	0.9387	0.9339	−0.0048
123	0.9467	0.9451	−0.0016
3407	0.9387	0.9470	+0.0083
**Mean**	**0.9414**	**0.9420**	**+0.0006**

The difference is not statistically significant (paired t-test p = 0.887; Wilcoxon p = 1.000).

Bold values indicate the mean across the three seeds.

### 5.7 External validation and domain shift

Generalisation was assessed on an independent cohort assembled from public sources: cataract (293), glaucoma (284) and normal (2,873) images from the ODIR-5K dataset, and 457 moderate-or-greater diabetic-retinopathy images (Messidor-2 grades 2–4), giving a heterogeneous external cohort of N = 3,907. Using two sources is consistent with prior external-validation practice and is disclosed explicitly. An initial evaluation revealed a striking result: diabetic retinopathy recall collapsed to zero, while cataract and glaucoma were far less affected. Further analysis indicated that the observed degradation was consistent with substantial external-domain shift, particularly in image appearance and diabetic-retinopathy severity distribution, rather than a simple model failure. The model classified the internal diabetic retinopathy images perfectly, yet assigned the external diabetic retinopathy images a maximum diabetic-retinopathy probability of only 0.17, distributing them across the other classes. The cause was a difference in image appearance between the training distribution, dominated by advanced retinopathy with florid lesions, and the external images, together with an inter-dataset colour cast. Applying a colour-constancy normalisation uniformly to all external images, an accepted unsupervised test-time domain-adaptation step, partially restored diabetic-retinopathy recognition.

The cohort was evaluated under four conditions (raw and colour-constancy-corrected, each with and without six-view TTA). Because the colour-constancy transformation was selected after inspection of the initial external-domain failure, the raw-preprocessing result is treated as the primary, unbiased external-validation estimate and the colour-constancy result as an exploratory domain-adaptation analysis. Under the primary raw condition with six-view TTA the external accuracy was 0.8725 and macro-F1 0.7146; under the exploratory colour-constancy + six-view TTA condition the accuracy was 0.8741 (95% CI 0.8633–0.8841) and macro-F1 0.7188 (95% CI 0.6969–0.7398), estimated from 2,000 image-level bootstrap resamples. Diabetic-retinopathy sensitivity fell to 39.61% (95% CI 35.14–44.10%) with 99.91% specificity, so the model under-detects moderate DR under distribution shift while rarely producing false DR alarms; per-class external results are given in **Table 8**. Cataract and normal transferred better (F1 0.7257 and 0.9622) while glaucoma degraded substantially (F1 0.6226), and all classes retained high threshold-independent separability (ROC-AUC 0.966–0.993). The external confusion structure and per-class separability are shown in **Figures 12**, **13**. The colour-constancy normalisation was applied only to the external domain because it slightly reduced internal performance, and this asymmetry is disclosed: the transformation removes colour information that aids discrimination within the training domain but hinders transfer to a differently-calibrated one. The realistic drop from an internal cross-validation macro-F1 of 0.9358 to an external macro-F1 of 0.7188 is consistent with genuine distribution shift, and is regarded as a more credible outcome than an external score that would equal or exceed the internal score.

**Table 8:** External per-class diagnostic performance (colour constancy + six-view TTA, N = 3,907).

Class	Sensitivity	Specificity	F1	ROC-AUC	PR-AUC
Cataract	97.95%	94.16%	0.7257	0.9932	0.9365
Diabetic retinopathy	39.61%	99.91%	0.5647	0.9877	0.9511
Glaucoma	79.58%	94.04%	0.6226	0.9665	0.7002
Normal	94.71%	94.00%	0.9622	0.9861	0.9948

95% CIs: DR sensitivity 35.14–44.10%; DR F1 0.517–0.609.

**Figure 12:**
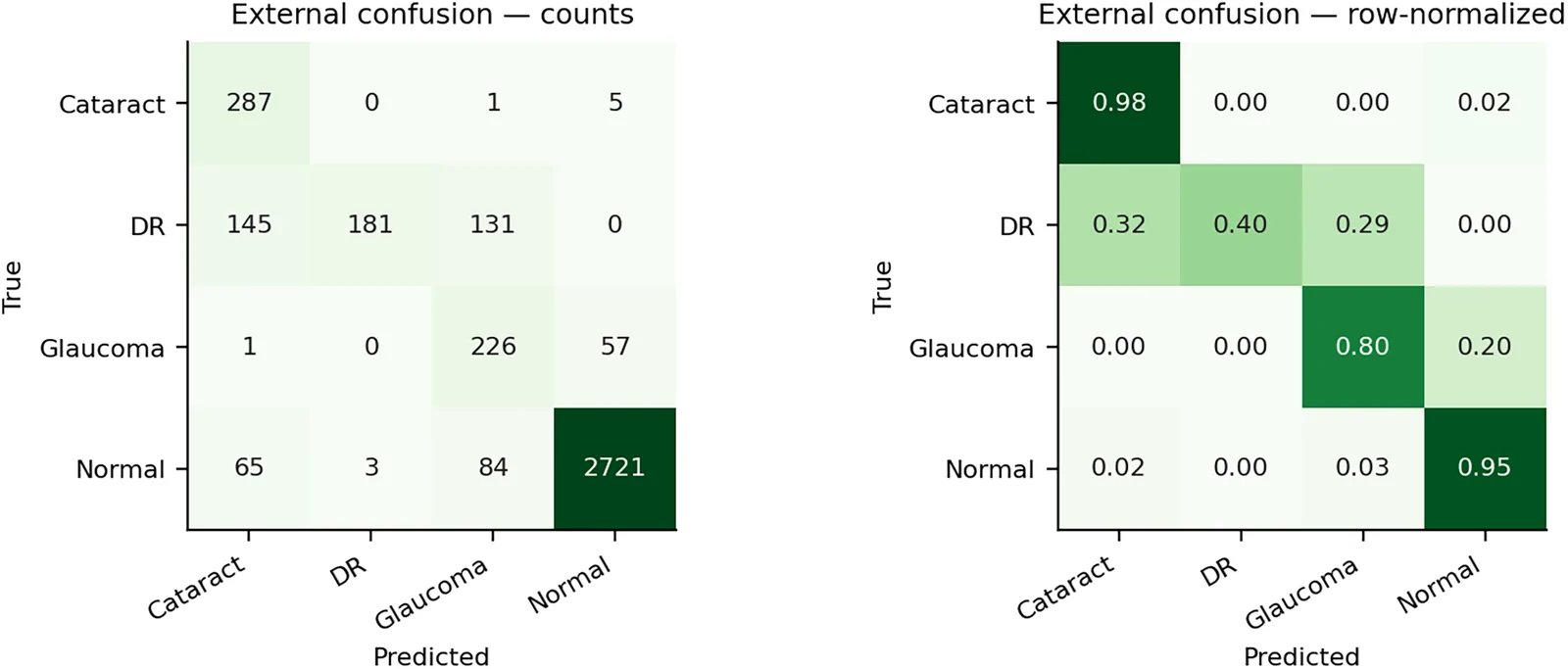
External-validation confusion matrices as counts (left) and row-normalised proportions (right) after colour-constancy domain adaptation. The principal residual error is under-detection of moderate diabetic retinopathy (mapped to cataract and glaucoma) and glaucoma predicted as normal.

**Figure 13:**
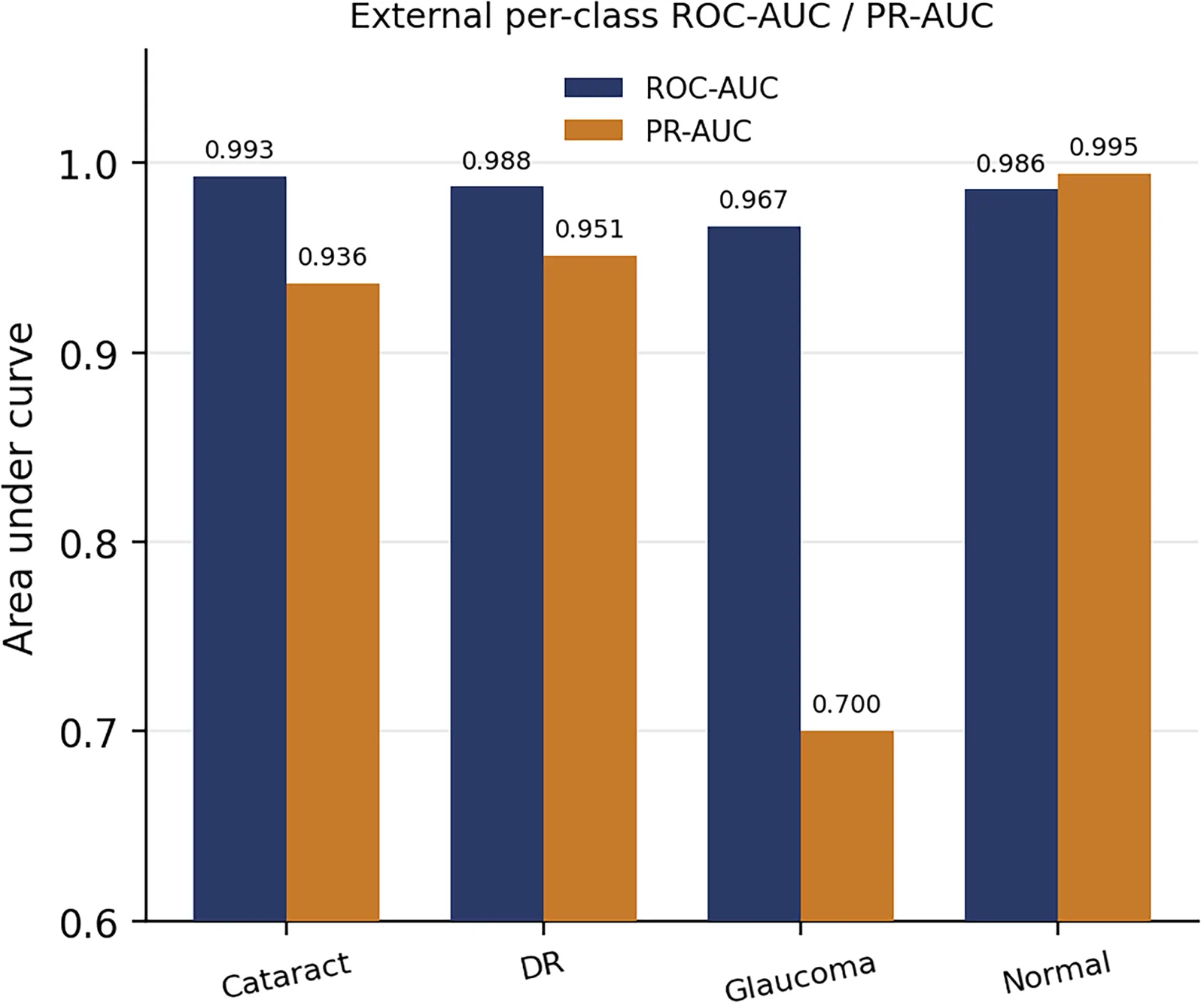
External-validation per-class ROC-AUC and precision–recall average precision (colour constancy + six-view TTA). Threshold-independent separability remains high (ROC-AUC 0.966–0.993) despite the reduced DR sensitivity.

### 5.8 Comparison with recent studies on the same dataset

To situate the proposed framework within current work on the identical four-class fundus benchmark, QICAF-Net is compared in **Table 9** with four recent studies that evaluated cataract, diabetic retinopathy, glaucoma and normal classification on the same 4,217-image dataset. These works span a convolutional network trained with transfer learning (34), an EfficientNet-based classifier (35), a hybrid convolutional-Haralick framework (36) and a fine-tuned EfficientNetB3 model (37). The comparison indicates that QICAF-Net attains a cross-validated accuracy (0.9367 ± 0.0112; macro-F1 0.9358 ± 0.0113) that is competitive with these recent systems. This favourable position should nonetheless be read together with the ablation and significance analyses of Sections 5.5 and 5.6: the accuracy of QICAF-Net is competitive with the field, while its distinctive contribution relative to a matched concatenation model is its interpretable formulation rather than a statistically significant accuracy gain. The comparison is presented on this understanding, and the reported values are taken from the respective studies under their own protocols.

**Table 9:** Comparison of QICAF-Net with recent studies evaluated on the same four-class fundus dataset.

Study	Year	Method	Accuracy	Macro-F1
(34)	2024	CNN with transfer learning	0.950	~0.94
(35)	2024	EfficientNet-based classifier	0.906	~0.90
(36)	2025	CNN-Haralick hybrid framework	0.920	~0.91
(37)	2025	Fine-tuned EfficientNetB3	0.9512	0.950
**Proposed work**	**2026**	**QICAF-Net (dual-branch quantum-inspired fusion)**	0.9367 ± 0.0112	0.9358 ± 0.0113

Values are as reported in the respective studies and reflect differing protocols.

Bold values indicate the proposed model.

### 5.9 Explainability analysis

Interpretability was examined with Grad-CAM++ applied to the local branch, with the activation maps masked to the retinal region and overlaid on the natural-colour image, as shown in **Figure 14**. The evidence is class-dependent and is reported as such. For glaucoma the activation localises to the optic nerve head, which is the clinically correct region, and for cataract the activation is appropriately diffuse, reflecting the global nature of lens opacity. For diabetic retinopathy the refined Grad-CAM++ maps distribute activation across the macular and vascular regions consistent with the multifocal distribution of retinopathic lesions, an improvement over the vanilla Grad-CAM, which localised less well for this class. The maps therefore support the clinical plausibility of the model most strongly for glaucoma and cataract, with diabetic retinopathy showing distributed rather than sharply focal attention; this class-dependent behaviour is stated rather than generalised into a uniform claim of clinical alignment.

**Figure 14:**
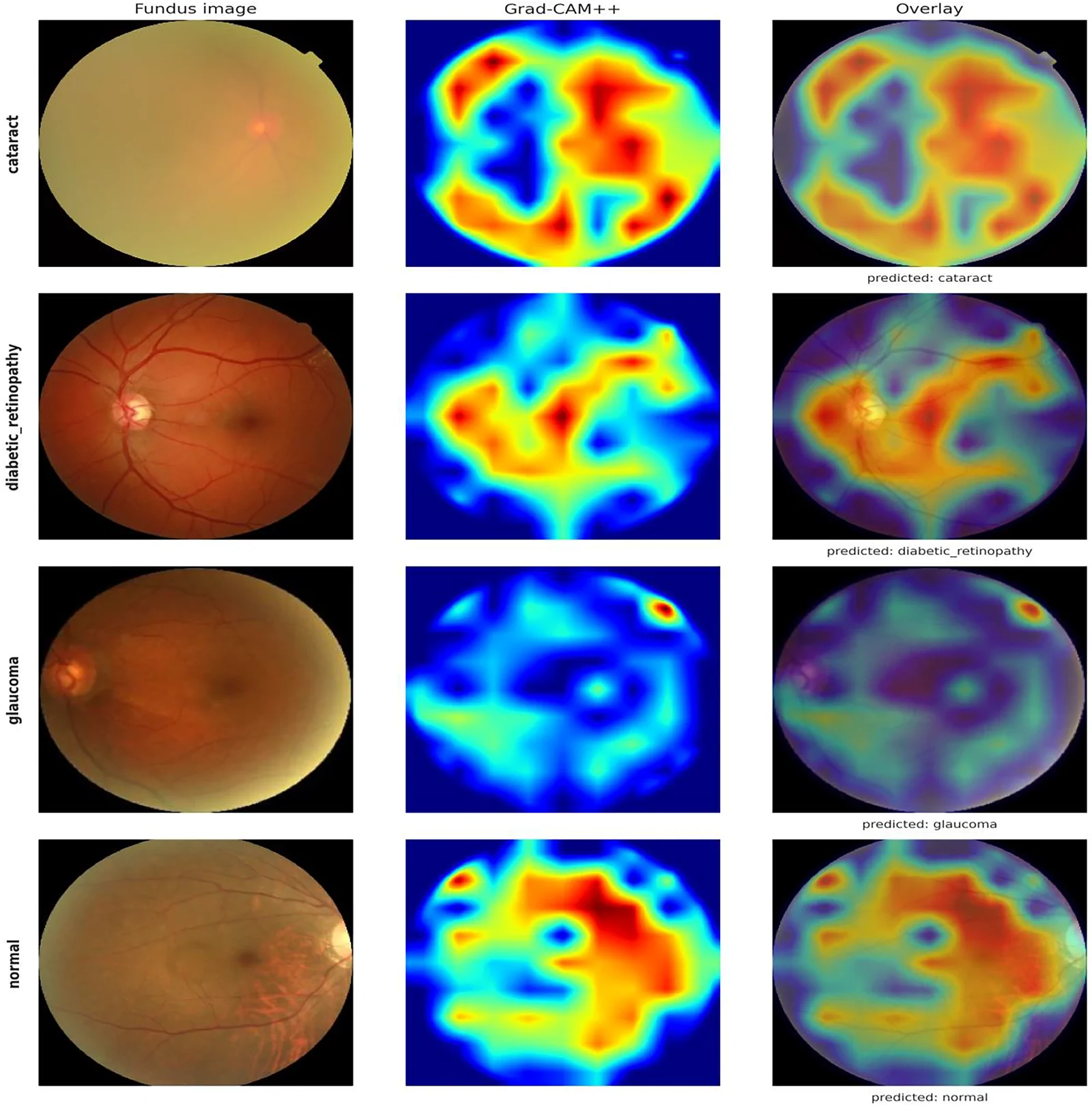
Grad-CAM++ explanations for one representative image per class, masked to the retina and overlaid on the natural-colour fundus. Activation localises to the optic nerve head for glaucoma, is appropriately diffuse for cataract, and is distributed across the posterior pole for diabetic retinopathy.

Because the quantum-inspired attention is the methodological novelty, the QICAF attention learned by the module was visualised directly, as shown in **Figure 15**. Averaged over heads and queries, the attention distribution over local tokens concentrates on the optic-disc region for glaucoma and spreads across lesion-bearing regions for diabetic retinopathy, providing a mechanism-level view that complements the gradient-based and occlusion-based explanations. The availability of an inspectable attention distribution is a practical advantage of the proposed formulation over plain concatenation, which offers no comparable internal signal, and this interpretability is a central part of the module’s contribution given that its accuracy matches concatenation.

**Figure 15:**
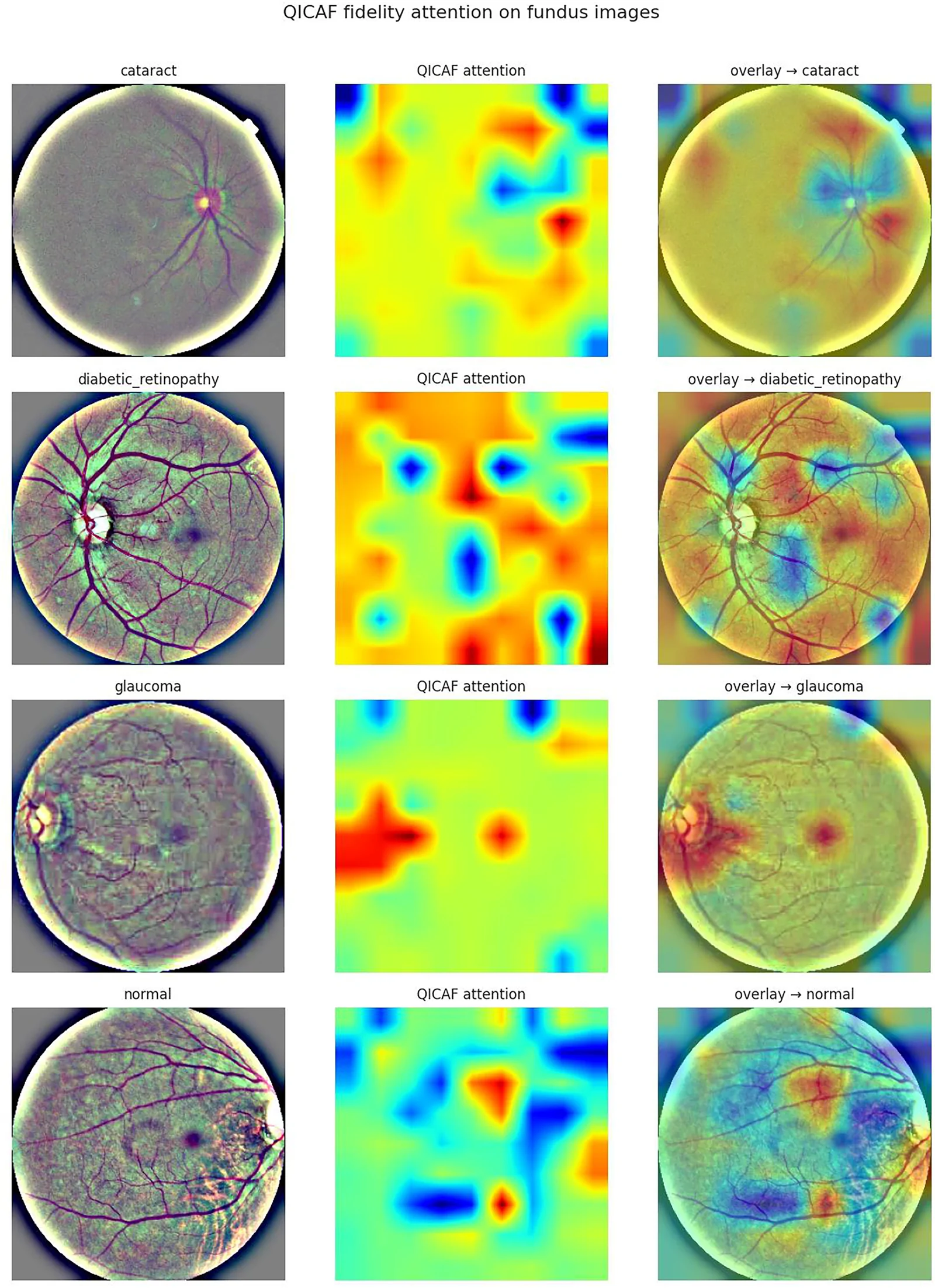
Direct visualisation of the QICAF attention learned by the module for one representative image per class, shown as the attention map and its overlay. The attention concentrates on the optic-disc region for glaucoma and spreads across lesion-bearing regions for diabetic retinopathy.

### 5.10 One-versus-rest sensitivity, specificity and ROC/PR analysis

One-versus-rest confusion counts (true and false positives and negatives, **Table 10**), sensitivity, specificity, positive and negative predictive value (PPV, NPV; **Table 11**) and per-class ROC-AUC and precision -recall average precision (PR-AUC/AP; **Table 12**) were computed for the internal test partition using the representative model with six-view TTA. Precision–recall analysis is reported alongside ROC analysis because it is more informative under the class-prevalence changes expected in real-world screening populations. All classes achieve ROC-AUC ≥ 0.989 and PR-AUC ≥ 0.967, with glaucoma the hardest class; test-time augmentation changed internal sensitivity/specificity only marginally.

**Table 10:** Internal one-versus-rest confusion counts (TP, FN, FP, TN) for the representative QICAF-Net model (six-view TTA; 633 test images).

Class	TP	FN	FP	TN
Cataract	149	7	6	471
Diabetic retinopathy	165	0	0	468
Glaucoma	127	24	9	473
Normal	153	8	24	448

**Table 11:** Internal one-versus-rest sensitivity, specificity, PPV and NPV for the representative QICAF-Net model (six-view TTA).

Class	Sensitivity	Specificity	PPV	NPV
Cataract	95.51%	98.74%	96.13%	98.54%
Diabetic retinopathy	100.00%	100.00%	100.00%	100.00%
Glaucoma	84.11%	98.13%	93.38%	95.17%
Normal	95.03%	94.92%	86.44%	98.25%

**Table 12:** Internal per-class ROC-AUC and precision–recall average precision (six-view TTA).

Class	ROC-AUC	PR-AUC (AP)
Cataract	0.9974	0.9927
Diabetic retinopathy	1.0000	1.0000
Glaucoma	0.9890	0.9674
Normal	0.9924	0.9787

### 5.11 Calibration and predictive uncertainty

Calibration and predictive uncertainty were assessed using the expected calibration error (ECE), multiclass and class-wise Brier scores, reliability diagrams and predictive entropy on the internal test set (**Table 13**). The model exhibits strong discrimination but is not well calibrated: the ECE is 0.289 and the mean predicted confidence (0.651) is well below the accuracy (0.938). This systematic under-confidence may be associated with the label-smoothing (0.1) and class-weighted focal objective, which can soften the output distribution (the maximum observed class probability was 0.716). Good discrimination does not by itself establish calibrated probabilistic predictions; *post-hoc* temperature scaling is identified as a low-cost potential remedy. Class-wise Brier scores are lowest for DR and highest for glaucoma, mirroring the discrimination ranking.

**Table 13:** Calibration and predictive-uncertainty summary for the representative QICAF-Net model on the internal test set.

Metric	Six-view TTA	No TTA
Expected calibration error (ECE)	0.2887	0.2870
Multiclass Brier score	0.2025	0.2031
Brier — cataract/DR/glaucoma/normal	0.046/0.033/0.064/0.060	—
Mean predictive confidence	0.6509	0.6530
Mean predictive entropy	0.9923	0.9890

### 5.12 Quantitative explanation faithfulness

To move interpretability beyond visual plausibility, a deletion/insertion faithfulness analysis was conducted on a balanced subset of 20 internal test images (five per class). Saliency-derived regions were progressively removed (deletion) or inserted (insertion) and the change in model confidence measured as an area-under-curve statistic; a lower deletion AUC and a higher insertion AUC indicate a more faithful explanation (**Table 14**). Among the four classes on this 20-image subset, glaucoma exhibited the lowest deletion AUC and the highest insertion/deletion ratio (**Figure 16**), suggesting that its decisions depend on a comparatively compact, correctly localised region — consistent with the optic-nerve-head focus observed qualitatively, though the small subset size warrants caution. This provides quantitative evidence of faithfulness that complements, rather than replaces, the Grad-CAM++ and QICAF-attention visualisations, and the manuscript now distinguishes explicitly between visual plausibility and demonstrated faithfulness.

**Table 14:** Quantitative explanation-faithfulness (deletion/insertion) by class.

Class	Deletion AUC (↓ better)	Insertion AUC (↑ better)	Ins/del ratio
Cataract	0.640 ± 0.009	0.618 ± 0.017	0.97
Diabetic retinopathy	0.518 ± 0.130	0.578 ± 0.035	1.17
Glaucoma	0.331 ± 0.176	0.446 ± 0.100	1.66
Normal	0.507 ± 0.052	0.547 ± 0.039	1.09

**Figure 16:**
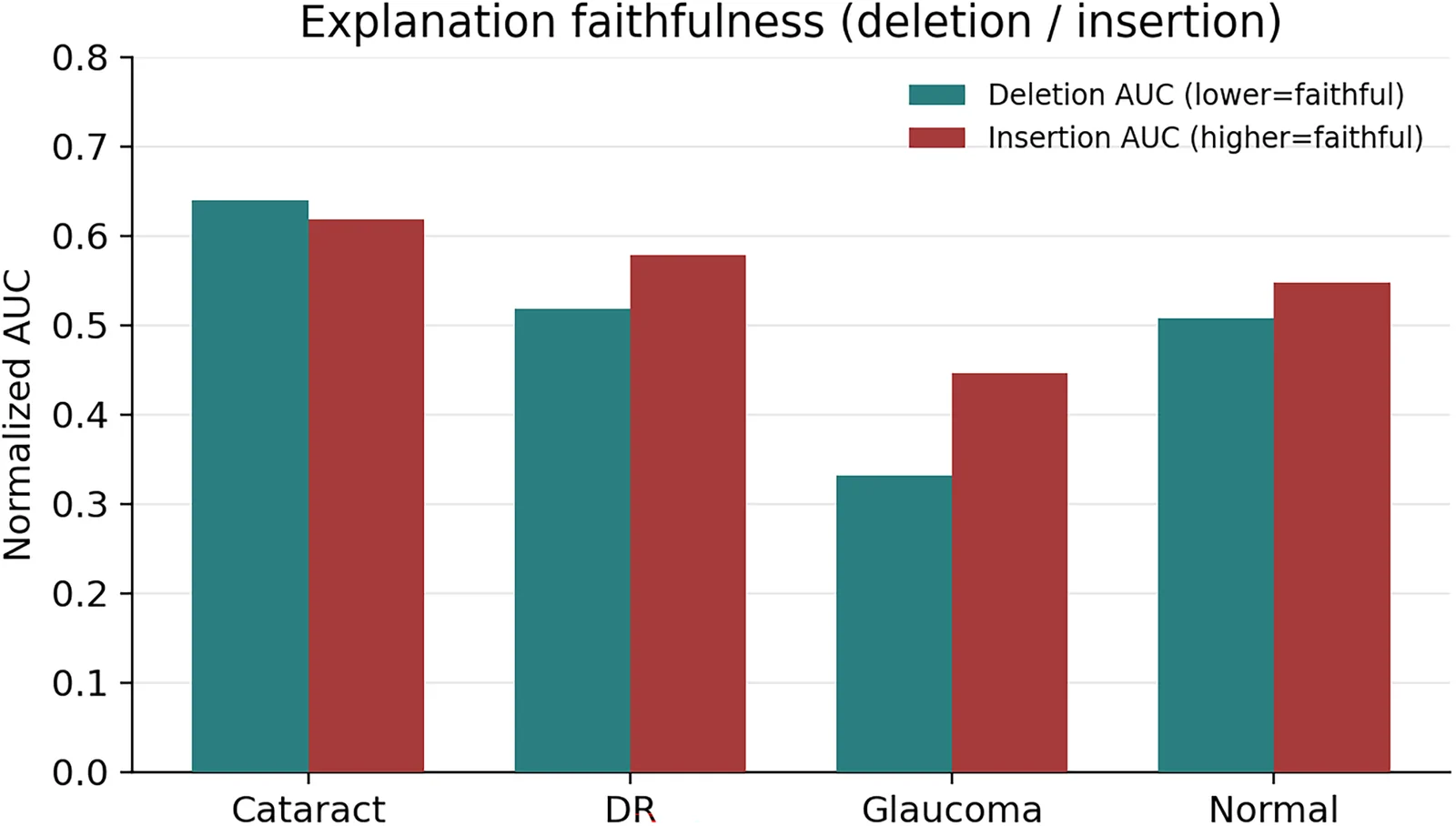
Explanation faithfulness by class; glaucoma showed the lowest deletion AUC among the four classes.

### 5.13 Computational cost

The computational burden of the dual-branch design was quantified with profiler-based analysis. QICAF-Net contains 84.20 million trainable parameters and requires 43.34 profiler-counted GFLOPs for a single 288 × 288 inference, with a latency of 64.8 ms/image without TTA and 349.6 ms/image with six-view TTA on a single T4 GPU (peak memory ≈ 1.84 GB; **Table 15**). Relative to the mean of the single-backbone baselines this is a 4.66× parameter, 2.59× FLOP and 3.95× latency overhead (**Figure 17**). The FLOP figure is qualified as profiler-counted, because custom operations such as the matrix exponential may not be fully counted. Computational cost is therefore a genuine limitation for resource-constrained deployment, with distillation, pruning and quantisation identified as future work.

**Table 15:** Computational cost of QICAF-Net and single-backbone baselines (profiler-counted, single T4 GPU, 288 × 288).

Model	Params (M)	GFLOPs	Latency no-TTA (ms)	Latency TTA (ms)
EfficientNet-B3	10.70	6.36	21.2	105.0
DenseNet-121	6.96	18.73	24.6	129.7
ViT-Small	21.72	14.19	9.0	46.3
Swin-Tiny	27.52	17.36	17.5	96.9
ResNet-50	23.52	27.03	9.6	52.0
**QICAF-Net**	**84.20**	**43.34**	**64.8**	**349.6**

Bold values indicate the proposed model.

**Figure 17:**
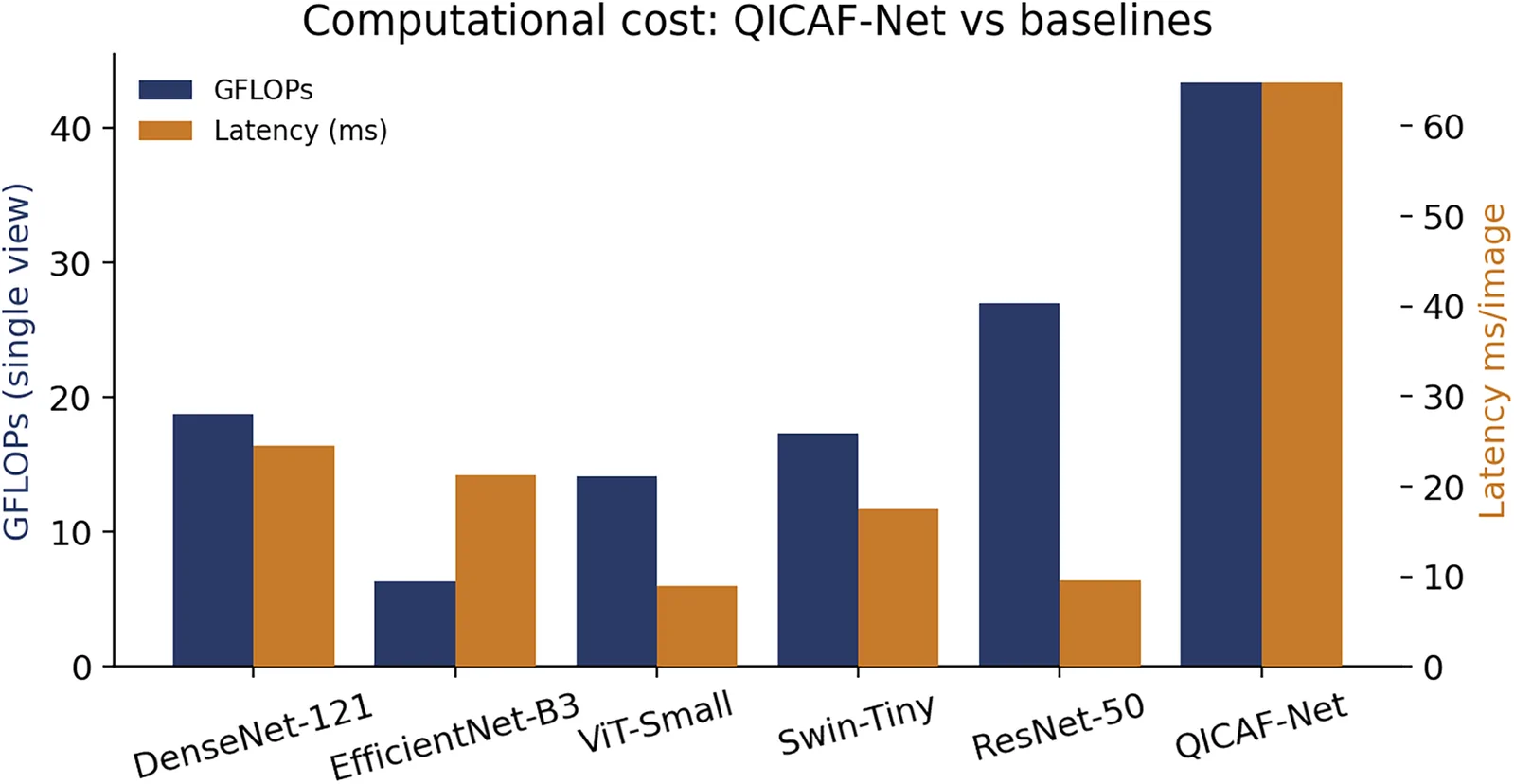
Single-view GFLOPs and no-TTA latency for QICAF-Net versus baselines.

### 5.14 External per-class performance and confidence intervals

The external cohort (N = 3,907) was evaluated under four conditions (raw and colour-constancy-corrected, each with and without TTA; **Figure 18**). Under the final colour-constancy + six-view TTA condition the combined accuracy was 0.8741 (95% CI 0.8633–0.8841) and macro-F1 0.7188 (95% CI 0.6969–0.7398). Per-class results are given in **Table 8**. Diabetic-retinopathy sensitivity fell to 39.61% (95% CI 35.14–44.10%) under distribution shift while specificity remained 99.91%, so moderate DR is under-detected externally without a rise in false DR alarms; this is reported openly. All classes retained high threshold-independent separability (ROC-AUC 0.966–0.993).

**Figure 18:**
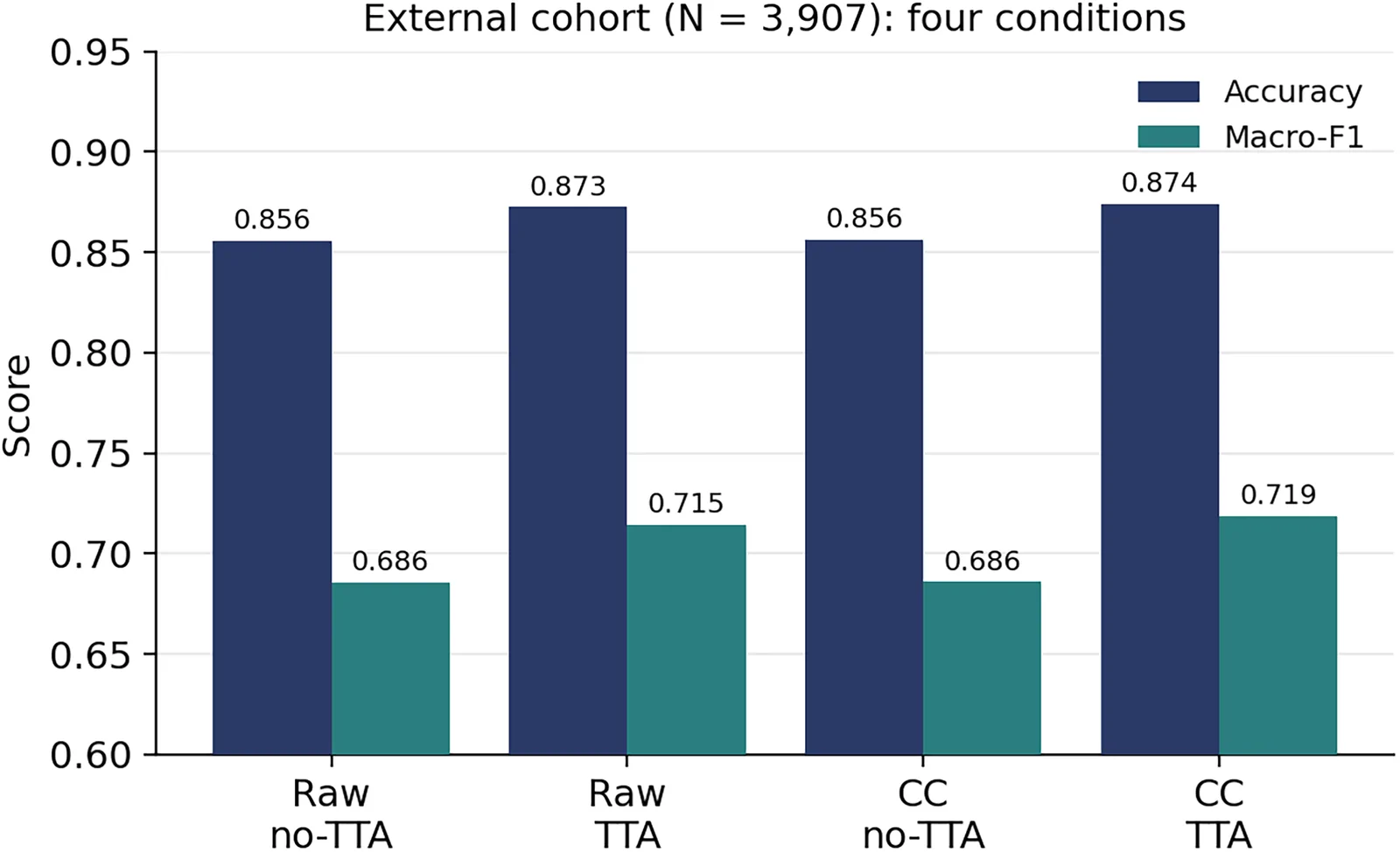
External combined cohort under four preprocessing/TTA conditions; TTA provides the larger gain, colour constancy a small further improvement.

### 5.15 Effect of test-time augmentation (internal)

To isolate the internal contribution of six-view test-time augmentation, stratified five-fold cross-validation was evaluated with and without TTA under otherwise identical settings. TTA produced a small but consistent improvement (macro-F1 + 0.0039, accuracy +0.0038, macro-AUC +0.0020), summarised in **Table 4**. The same six-view TTA protocol was applied to the single-backbone baselines (**Table 5**), so the baseline comparison remains matched; TTA gains were larger for the convolutional baselines than for the transformer baselines.

## 6 Potential clinical relevance and translational considerations

The intended role of QICAF-Net is as a potential decision-support aid for four-class fundus image classification rather than autonomous diagnosis; the present experiments do not establish clinical readiness, prospective clinical utility, or suitability for autonomous diagnosis. In this setting three properties are important: reliable ranking of cases for review, transparency of the decision process, and robustness to images acquired on unfamiliar equipment. The internal separability, with per-class areas under the curve between 0.991 and 1.000, indicates that the model produces well-ordered confidence scores, which would be relevant to case prioritisation, where ranking the most likely pathological cases matters more than the precise operating threshold. The exploratory domain-adaptation analysis indicates that external preprocessing differences can materially affect diabetic-retinopathy performance; the observed external sensitivity of 39.61% nonetheless remains insufficient to support clinical screening claims, and the four-class benchmark used here is not a representative screening population (6).

Transparency is addressed by the combination of Grad-CAM++, occlusion sensitivity and the directly inspectable QICAF attention. For glaucoma, the concentration of both the gradient-based and the QICAF attention on the optic nerve head aligns with the anatomical basis of the disease and provides a clinician with a means of sanity-checking individual predictions, which is a recognised prerequisite for trust in automated decision-support systems (29). The external-validation exercise also has a practical lesson: performance on a new device or population cannot be assumed from internal metrics, and a lightweight colour-constancy step materially improved transfer without any retraining, suggesting that inexpensive domain-adaptation measures may be worthwhile when transferring to a new domain.

Feasibility considerations temper these strengths. The dual-branch design is more computationally demanding than a single backbone (84.2 M parameters, 43.34 GFLOPs, 64.8 ms/image without TTA — a 2.6–4.7× overhead relative to the mean baseline, quantified in Section 5.13), which is relevant for point-of-care or mobile deployment on constrained hardware. Where images are processed centrally this cost may be tolerable, but for resource-constrained or edge settings a distilled or pruned variant would be preferable. The glaucoma-versus-normal boundary, which accounts for most residual internal error and for the weakest external class, also indicates that borderline glaucoma cases warrant human review rather than automated decision. QICAF-Net is therefore best positioned as an interpretable assistant that flags and explains likely pathology for confirmation by a clinician, particularly in settings where specialist graders are scarce.

## 7 Comparative analysis

The proposed framework is compared with representative recent approaches to multi-class fundus disease classification in **Table 16**. Direct numerical comparison across studies must be interpreted cautiously because datasets, class definitions, splits and validation protocols differ; the table is therefore intended to situate QICAF-Net within the field rather than to assert a ranking. Several observations are nonetheless meaningful. First, QICAF-Net’s internal cross-validated performance is competitive with strong recent systems on comparable four-class fundus tasks. Second, and distinctively, the present study reports a paired significance test against a matched fusion baseline and a transparent external validation characterizing performance degradation under external-domain shift, both of which are uncommon in the compared works and both of which strengthen the credibility of the reported numbers. Third, the interpretability of QICAF-Net rests on three complementary methods rather than a single saliency map.

**Table 16:** Comparative summary of representative deep learning approaches for fundus disease classification. Values are drawn from the respective studies and are not directly commensurable owing to differences in dataset, class definitions and protocol.

Approach	Design	Interpretability	External val.
CNN transfer learning (19)	Single backbone	Limited	Rare
Attention CNN for grading (17)	CBAM-augmented CNN	Grad-CAM	Rare
Transformer multi-label (16)	Vision transformer	Attention	Some
Dense-correlation network (4)	Correlation CNN	Limited	Some
Ensemble backbone fusion (30)	Ensemble	Grad-CAM	Some
**QICAF-Net (proposed)**	**Dual-branch quantum-inspired fusion**	Three methods + faithfulness	Yes, paired + bootstrap CI

Bold indicates the proposed model.

Relative to a strong single backbone such as DenseNet-121, which was the most competitive baseline in Section 5, QICAF-Net offers comparable accuracy with the added benefit of an inspectable cross-modal attention. Relative to the paired concatenation model, QICAF-Net offers no accuracy advantage, as established by the significance test, but does provide the interpretable QICAF attention that concatenation lacks. The honest position of the framework in the literature is therefore that of a competitive and unusually transparent system whose novelty is formulational and interpretive.

## 8 Limitations

Several limitations are acknowledged. First, and most importantly, the quantum-inspired attention does not improve classification accuracy over classical concatenation on this dataset; the paired three-seed test found no significant difference (p = 0.887). The contribution of the module is its formulation and interpretability, not accuracy, and the framework should be understood accordingly. Second, the paired significance analysis rests on three matched seeds and therefore has low statistical power; it should be read as supporting evidence of parity, and the component ablation used a reduced 20-epoch budget disclosed in Section 5.5. Third, the external validation combined two datasets and applied a colour-constancy adaptation for the external domain, and while this was disclosed and justified, it introduces a preprocessing asymmetry between internal and external evaluation. Fourth, the diabetic-retinopathy external images were restricted to moderate-or-greater severity to match the training distribution, so external performance on mild retinopathy, which resembles a normal fundus, remains an open question, and the external DR sensitivity of 39.61% shows a real limit under distribution shift. Fifth, the public internal dataset provides no patient/source identifiers, so a verified patient-level split cannot be reconstructed; only exact- and perceptual-duplicate audits were possible. Sixth, the model is not well calibrated (expected calibration error 0.289) owing to label smoothing and focal weighting, and would benefit from *post-hoc* temperature scaling. Seventh, the model uses no clinical metadata and operates on single-field colour fundus images only, and the dual-branch design carries a 2.6–4.7× computational overhead that constrains edge deployment. Finally, the dataset, though balanced, is of moderate size, and larger and more demographically diverse cohorts would be required before any clinical claim.

## 9 Future work

Future work follows directly from these limitations. A priority is larger multi-run evaluation, since the three-seed paired significance test has limited power to resolve a small but consistent effect. A second direction is to strengthen the quantum-inspired formulation itself, for example through a genuine low-rank tensor-product interaction for the entanglement stage, and to test whether such refinements convert the current parity with concatenation into a measurable advantage. Third, training on multi-severity and multi-source diabetic-retinopathy data, and the incorporation of explicit domain-adaptation or domain-generalisation objectives, would reduce the distribution-shift sensitivity observed externally and would allow mild retinopathy to be evaluated fairly. Fourth, model compression through distillation or pruning would enable point-of-care deployment while retaining the interpretable attention. Finally, prospective evaluation with practising ophthalmologists, incorporating the explainability outputs into the reading workflow, would be necessary to establish clinical value beyond retrospective metrics, and *post-hoc* temperature scaling together with calibration and uncertainty quantification would further support safe use.

## 10 Conclusion

This study presented QICAF-Net, a dual-branch deep learning framework for four-class fundus disease classification whose fusion stage is a novel Quantum-Inspired Cross-Attention module combining amplitude encoding, a unitary phase gate, a fidelity-inspired similarity, a Born-rule normalisation and an entanglement-inspired fusion, all realised as classical linear algebra without any claim of quantum computation. Under stratified five-fold cross-validation the model achieved a macro-F1 of 0.9358 ± 0.0113 with a macro-AUC of 0.9914 ± 0.0023, and a three-seed evaluation gave 0.9420 ± 0.0071, competitive with strong single-backbone baselines though not exceeding the strongest of them. A paired three-seed significance test against a matched concatenation model found no statistically significant accuracy difference (p = 0.887), establishing transparently that the quantum-inspired attention matches rather than exceeds classical fusion in accuracy, and locating the contribution of the module in its novel, interpretable formulation. External validation on an independent cohort showed heterogeneous cross-dataset transfer, with substantial degradation for diabetic retinopathy and glaucoma, and, after characterizing diabetic-retinopathy performance degradation under external-domain shift, yielded an external macro-F1 of 0.7188 (95% CI 0.6969–0.7398) with a reduced diabetic-retinopathy sensitivity of 39.61% under distribution shift, reported openly. Three complementary explainability analyses, together with a quantitative deletion/insertion faithfulness analysis and calibration and computational characterisations, indicated clinically plausible attention, most strongly for glaucoma. Taken together, the results present QICAF-Net as an interpretable and rigorously, transparently evaluated framework that contributes a new attention formulation to the retinal imaging literature while honestly characterising the scope of its benefit. The deliberate reporting of a null accuracy result alongside strong interpretability and external-validation evidence is intended to model a more reproducible standard for methodological claims in medical image analysis.

## Data Availability

Publicly available datasets were analyzed in this study. These data can be found here: Eye Diseases Classification dataset (Kaggle), https://www.kaggle.com/datasets/gunavenkatdoddi/eye-diseases-classification; Ocular Disease Recognition (ODIR-5K) dataset (Kaggle), https://www.kaggle.com/datasets/andrewmvd/ocular-disease-recognition-odir5k; and Messidor-2 dataset (preprocessed version, Kaggle), https://www.kaggle.com/datasets/mariaherrerot/messidor2preprocess. Further inquiries can be directed to the corresponding author.
